# W196 and the *β*-Hairpin Motif Modulate the Redox Switch of Conformation and the Biomolecular Interaction Network of the Apoptosis-Inducing Factor

**DOI:** 10.1155/2021/6673661

**Published:** 2021-01-15

**Authors:** Silvia Romero-Tamayo, Ruben Laplaza, Adrian Velazquez-Campoy, Raquel Villanueva, Milagros Medina, Patricia Ferreira

**Affiliations:** ^1^Departamento de Bioquímica y Biología Molecular y Celular, Facultad de Ciencias, Universidad de Zaragoza, Spain; ^2^Instituto de Biocomputación y Física de Sistemas Complejos, BIFI (GBsC-CSIC and BIFI-IQFR Joint Units), Universidad de Zaragoza, Spain; ^3^Sorbonne Université, CNRS, Laboratoire de Chimie Théorique, LCT, 75005 Paris, France; ^4^Departamento de Química Física, Universidad de Zaragoza, 50009 Zaragoza, Spain; ^5^Fundación ARAID, Diputación General de Aragón, Spain; ^6^Aragon Institute for Health Research (IIS Aragon), Zaragoza, Spain; ^7^Biomedical Research Networking Centre for Liver and Digestive Diseases (CIBERehd), Madrid, Spain

## Abstract

The human apoptosis-inducing factor (hAIF) is a moonlight flavoprotein involved in mitochondrial respiratory complex assembly and caspase-independent programmed cell death. These functions might be modulated by its redox-linked structural transition that enables hAIF to act as a NAD(H/^+^) redox sensor. Upon reduction with NADH, hAIF undergoes a conformational reorganization in two specific insertions—the flexible regulatory C-loop and the 190-202 *β*-harpin—promoting protein dimerization and the stabilization of a long-life charge transfer complex (CTC) that modulates its monomer-dimer equilibrium and its protein interaction network in healthy mitochondria. In this regard, here, we investigated the precise function of the *β*-hairpin in the AIF conformation landscape related to its redox mechanism, by analyzing the role played by W196, a key residue in the interaction of this motif with the regulatory C-loop. Mutations at W196 decrease the compactness and stability of the oxidized hAIF, indicating that the *β*-hairpin and C-loop coupling contribute to protein stability. Kinetic studies complemented with computational simulations reveal that W196 and the *β*-hairpin conformation modulate the low efficiency of hAIF as NADH oxidoreductase, contributing to configure its active site in a noncompetent geometry for hydride transfer and to stabilize the CTC state by enhancing the affinity for NAD^+^. Finally, the *β*-hairpin motif contributes to define the conformation of AIF's interaction surfaces with its physiological partners. These findings improve our understanding on the molecular basis of hAIF's cellular activities, a crucial aspect for clarifying its associated pathological mechanisms and developing new molecular therapies.

## 1. Introduction

**Figure 1:**
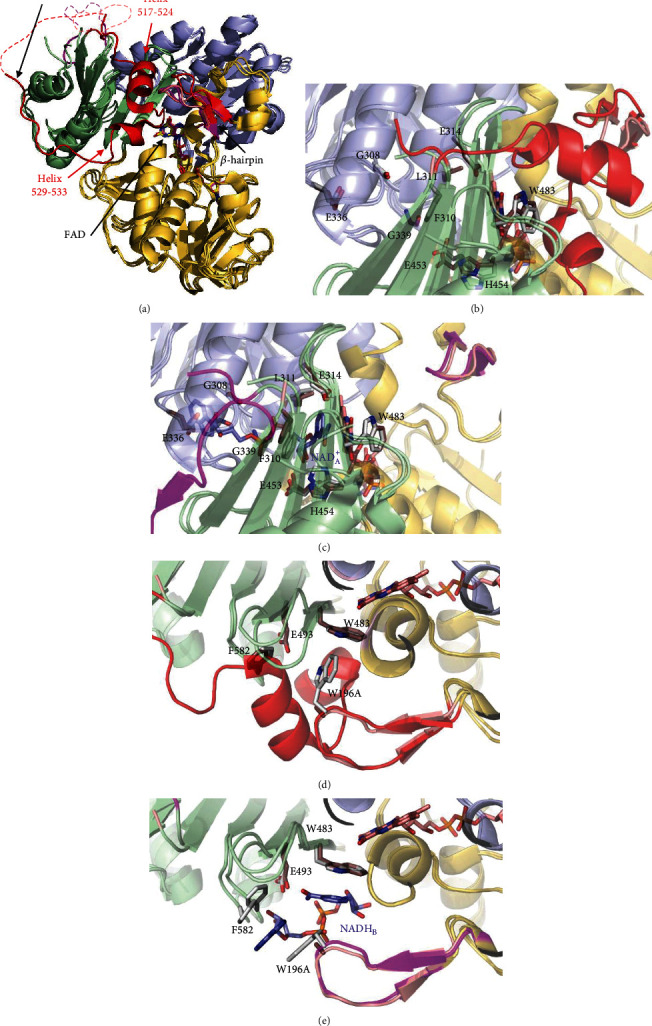
Comparative overview of the crystallographic structures of WT hAIF_*Δ*1-101ox_ (PDB 4BV6), WT CTC hAIF_*Δ*1-101rd_:2NAD(^+^/H) (PDB 4BUR), and W196A_ox_ variant (PDB 5KVH). (a) Cartoon superposition. FAD-, NADH-, and C-terminal domains colored in gold, light blue, and pale green, respectively. FAD drawn as sticks with C atoms in yellow, salmon, and magenta, respectively, for WT_ox_, WT CTC, and W196A_ox_ structures. Visible residues in the *β*-hairpin and the regulatory 509-560 C-loop are shown in red, magenta, and salmon, respectively, for WT_ox_, WT CTC, and W196A_ox_ structures. Missing fragments of the C-loop (P545-D559, K518-G557 and A511-D559 in WT_ox_ chain A, WT CTC chain C, and W196A_ox_ chain A structures, respectively) are indicated as dashed lines. Detail of the W196A_ox_ NADH_A_ binding site overlaid with (b) WT_ox_ and (c) WT CTC. Detail of the W196A_ox_ NADH_B_ binding site overlaid with (d) WT_ox_ and (e) WT CTC. Side chains for relevant residues are shown as CPK colored sticks with C atoms in salmon for W196A_ox_ and in light grey for WT_ox_ and WT CTC structures. NAD(^+^/H)_A_ and NADH_B_ in the WT CTC structure are shown as CPK colored sticks with its C atoms in blue.

The human apoptosis-inducing factor (hAIF) was first described as a mitochondrial-released flavoprotein mediating caspase-independent programmed cell death [1]. Moreover, this ubiquitously expressed protein across eukaryotes also plays a vital role in cell development and survival [2]. These survival functions rely on its FAD-dependent activities, which contribute to maintain the stability of the mitochondrial electron transfer chain, supercomplex organization, and transmembrane potential, as well as to control mitochondrial reactive oxygen species (ROS) [3]. In healthy mitochondria, the hAIF is processed and the hAIF_*Δ*1-53_ mature protein anchors in the inner membrane (IM)—*via* its N-terminal segment, facing the intermembrane space (IMS)— and folds in three domains (Figure 1(a)) [4–6]. Mammalian AIFs have two specific insertions, a regulatory C-terminal loop (aa 510-560 in hAIF) and a *β*-hairpin (aa 190-202 in hAIF), which connect the NADH and FAD domains to the C-terminal proapoptotic domain (Figure 1(a)).

hAIF conformation is dynamically influenced by coenzyme substrate binding and by the redox switch of its flavin cofactor, facts believed to modulate its biomolecular interaction network [7, 8]. In oxidized hAIF (hAIF_ox_), the regulatory C-loop is stabilized in the protein core by direct interaction with the *β*-hairpin, particularly through stacking and H-bonding interactions of W196 and R201 residues with its 517-524 and 529-533 short helixes. Binding of one NADH molecule to AIF's active site (NADH_A_) promotes FAD reduction, as well as the stabilization of a long-lived FADH^−^/NAD^+^ charge transfer complex (CTC). This CTC is inefficient in electron transfer, but capable of inducing a redox-linked protein conformational reorganization and its subsequent dimerization. CTC formation displaces the *β*-hairpin that triggers C-loop remodeling and its release to the solvent. These conformational changes induce (i) the allosteric formation of the second noncatalytic NADH binding site (NADH_B_), where stacking interactions with reoriented W196 and F582 side chains facilitate NADH_B_ accommodation and (ii) the dimerization of the protein (Figures 1(c) and 1(e)). These facts led to postulate AIF as a redox sensor of NAD(H/^+^) cellular levels [9–11]. W196 substitution by alanine disrupts the interaction between the *β*-hairpin and the C-loop that unwinds the above mentioned 529-533 helix and releases the two specific AIF insertions to the solvent, promoting a permissive mutant dimerization in its oxidized state (W196A hAIF_∆1-101ox_, herein W196A_ox_) (Figures 1(a) and 1(d)) [11]. However, W196A_ox_ maintains an active-site architecture similar to that of WT hAIF_ox_ for residues involved in NADH_A_ binding with the only exceptions of E453 and H454 **(**Figure 1(b)). The *β*-hairpin release in W196A_ox_ also induces the displacement of the central *β*-strand and the reorientation of E453 and H454 side chains (Figure S1D-E). Thus, H454 disrupts its interaction with S480, producing as a consequence the displacement of the H478 side chain—sited in the loop connecting the central *β*-strand and the His-rich helix—towards the C-loop, contributing to its release, and the exposition of the hydrophobic border at the dimerization interfaces in the W196A_ox_ structure. Such last conformational changes are similar to those reported for the WT CTC structure (Figure S1E-F).

In healthy cells, hAIF is essential for mitochondrial bioenergetics, being its physical and functional interaction with human CHCHD4 (coiled-coil-helix-coiled-coil-helix domain containing 4) key in the assembly and/or stabilization of multisubunit respiratory transport chain complexes and supercomplexes [12–15]. In IMS, CHCHD4 controls the import and oxidative folding of a set of assembly factors and protein subunits of respiratory complexes, while hAIF would regulate CHCHD4 expression as well as its import and proper IMS localization. Consequently, downregulation or depletion of hAIF gives rise to major dysfunctions in oxidative phosphorylation (OXPHOS), secondary to the deficiency of CHCHD4, causing severe neurodegenerative illnesses [12, 14, 16, 17]. The hAIF conformation—modulated by its redox NADH-dependent monomer-dimer equilibrium—is suggested to be critical for this interaction [7, 13, 18].

Upon lethal cellular stress, hAIF acts as a mediator of necrotic poly(ADP-ribose) polymerase- (PARP-) 1-dependent cell death (parthanatos) by its further processing into the soluble proapoptotic form (hAIF_*Δ*1-101_) and its release into the cytosol. The regulatory mechanism by which AIF is released is unknown, but could be somehow modulated by its structural reorganization due to depletion of coenzyme levels during PARP-1 hyperactivation [19]. Once in the cytosol, its interaction with some endonucleases, as cyclophilin A (CypA), favors nuclear cotranslocation of the AIF:CypA complex [20, 21]. In this subcellular compartment, the association of this binary complex to the histone H2AX leads to the assembly of the AIF-mediated DNA degradation complex (“degradosome,” AIF:CypA:H2AX:DNA), which provokes chromatin condensation and DNA fragmentation [21, 22].

Despite the emerging picture of the physiological functions of AIF being modulated by its conformational and redox states, we are only starting to depict the implications of the molecular mechanism regulating its activities. Thus, the molecular basis for the mechanism by which AIF regulates and pivots the redox-dependent interaction with CHCHD4, as well as those for the action of the degradosome complex as a death effector remain unknown. Nonetheless, we can envisage that AIF ability to stabilize both stable CTC and dimers—upon interaction with the coenzyme followed by FAD reduction—is surely a key feature to switch among its *in vivo* roles. In this context, the structural changes induced by CTC formation in native protein, but also shared by W196A_ox_, suggest that W196 and/or the *β*-hairpin might be relevant for AIF cellular activities. Such hypothesis is further supported by the *β*-hairpin contributing to binding of the allosteric NADH_B_, as well as by the fact that pathogenic mutations coursing with severe processes of neurodegeneration and early death have been reported at both the NADH_B_ binding site and the *β*-hairpin itself.

In the present study, we particularly investigate the contribution of the *β*-hairpin to the regulation of hAIF structural stability, coenzyme binding, reductase activity, CTC stability, and interaction with its physiological partners, by generating W196A, W196L, and W196Y site-directed mutants (which progressively reduce aromatic and stacking interactions). Our results indicate that the W196 side chain is not only key to establish the *β*-hairpin and C-loop organization in the oxidized state, but also to regulate the stability and conformational landscape of the protein. Both facts seem to be relevant to determine AIF efficiency as a cellular redox sensor, as well as to the establishment of specific binary interactions with different partners.

## 2. Materials and Methods

### 2.1. Expression and Production of Proteins

The cDNA sequences encoding for W196Y, W196L, and W196A hAIF_∆1-101_ variants (UniProtKB O95831) were obtained by site-directed mutagenesis from Mutagenex® and then subcloned into the pET28a expression vector with a cleavable N-terminal His_6_-tag similar to that reported for the WT protein [10]. The cDNAs encoding for human CypA (UniProtKB P62937), CHCHD4 (UniProtKB Q8N4Q1), and Histone H2AX (UniProtKB P16104) were synthetized with a cleavable N-terminal His_6_-tag (CACCAT) and codon optimized for *Escherichia coli* expression by GenScript®. The coding sequences were subcloned into the pET28a expression vector between two restriction sites: NdeI-NotI for CypA and CHCHD4 and NcoI-NdeI for H2AX. The resulting constructs were used to transform the *E. coli* C41 (DE3) strain for heterologous protein expression. Proteins were expressed and purified as described in the supplementary materials.

### 2.2. Molecular Weight Determination by Size Exclusion Chromatography

The hAIF_*Δ*1-101_ variants, either in the presence or absence of a 10-fold excess of NADH, were loaded onto a HiPrep 26/60 Sephacryl™S-200 High Resolution (*GE Healthcare*, Chicago, IL) column attached to a fast pressure liquid chromatographic system (*GE Healthcare*, Chicago, IL). Protein elution was performed in 50 mM phosphate buffer, 150 mM NaCl, pH 7.4, at a flow rate of 0.5 mL/min. The column was previously calibrated with the GE Healthcare LMW calibration kit (6 proteins in the 6400-160000 Da range). The obtained chromatograms were fitted to a set of Gaussian functions.

### 2.3. Stabilization of Cross-Linked Protein Oligomers and Electrophoretic Analysis

Reaction mixtures containing 4 *μ*M of the hAIF_∆1-101_ variants in 10 mM phosphate, pH 7.4 were incubated with a 100-fold excess of the homobifunctional-bis[sulfosuccinimidyl]-suberate (BS^3^) (Pierce) cross-linker at room temperature in the absence or presence of a 10-fold excess of NADH. Reactions were stopped by the addition of the denaturing bromophenol blue sample buffer and heated 5 min at 95°C. Sample mixtures were then resolved by 12% SDS-PAGE.

### 2.4. Spectroscopic Characterization

UV-visible spectra were recorded in a Cary 100 Bio spectrophotometer (*Agilent*, Santa Clara, CA). Protein concentrations were determined using the molar absorption coefficients of each variant, which were estimated by protein denaturation with 3 M guanidinium chloride in 10 mM phosphate, pH 7.4, followed by quantification of the released FAD. The extinction coefficients for WT, W196A, W196L, and W196Y hAIF_*Δ*1-101ox_ were *ε*_451nm_ = 13.7 M^−1^ cm^−1^ [10], *ε*_451nm_ = 13.35 M^−1^ cm^−1^, *ε*_451nm_ = 13.92 M^−1^ cm^−1^, and *ε*_452nm_ = 14.01 M^−1^ cm^−1^ respectively. Circular dichroism (CD) spectra were recorded in a thermostated Chirascan (*Applied Photophysics Ltd*., Surrey, UK). Far-UV CD spectra were acquired using 1 *μ*M protein in a 0.1 cm pathlength cuvette, while near-UV/Vis CD spectra were recorded using 20 *μ*M protein in a 1 cm pathlength cuvette. Fluorescence spectra were recorded in a thermostated Cary Eclipse Fluorescence spectrophotometer (*Agilent*, Santa Clara, CA) using 2 *μ*M protein in a 1 cm pathlength cuvette. Flavin fluorescence emission spectra were acquired in the 480-600 nm range upon excitation at 450 nm. Fluorescence emission spectra of aromatic residues were collected from 300 to 550 nm upon excitation at 280 nm. CD and fluorescence spectra were recorded in the absence and presence of a 100-fold excess of NADH at 10°C (folded state) and 90°C (thermally denatured state).

### 2.5. Thermal Denaturation Assays

Thermal denaturation curves were followed by changes in the FAD fluorescence emission upon its release from the protein by sample excitation at 450 nm. Curves were monitored from 10 °C to 90 °C with scan rates of 1°C/min, both in the absence and presence of a 100-fold excess of NADH. The curves for each variant were roughly normalized to values between 0 and 1 and globally fitted to a two-step process describing a single transition unfolding equilibrium (native (N)↔unfolded (U)) by using the following equation [23]:

(1) $$\begin{array}{c} S_{\text{obs}}=\frac{S_{N}+m_{N}T+\left(S_{U}+m_{U}T\right)e^{-\left(\Delta G/RT\right)}}{1+e^{-\left(\Delta G/RT\right)}}, \end{array}$$

in which *S*_obs_ is the measured protein signal at a given temperature (*T*). *S*_*N*_ and *S*_*U*_ are intercept at 0 K with the *y*-axis of the linear extrapolation for the native and unfolded pre- and posttransition regions, respectively, while *m*_*N*_ and *m*_*U*_ are the corresponding slopes. The stabilization Gibbs energy depends on temperature according to Δ*G* = Δ*H*(1 − 1/*T*_*m*_) + Δ*C*_*P*_(*T* − *T*_*m*_ − *T*ln(*T*/*T*_*m*_)), where Δ*H* is the unfolding enthalpy, *T*_*m*_ is the midtransition temperature, Δ*C*_*P*_ is the unfolding heat capacity change, and *R* is the ideal gas constant.

### 2.6. Kinetics Measurements

The steady-state diaphorase activity of hAIF_∆1-101_ variants was measured in air saturated 50 mM potassium phosphate, pH 8.0, using NADH as the substrate donor and 95 *μ*M dichlorophenolindophenol (DCPIP, Δ*ε*_620nm_ = 21 mM^−1^ cm^−1^) as acceptor [10]. When saturation profiles on the pyridine nucleotide concentration were observed, kinetic constants were estimated by fitting initial reaction rates at different coenzyme concentrations to the Michaelis-Menten equation:

(2) $$\begin{array}{c} \frac{\nu }{\text{e}}=\frac{k_{\text{cat}}\;\left[\text{NADH}\right]}{K_{m}^{\text{NADH}}+\left[\text{NADH}\right]},\\ \frac{\nu }{\text{e}}=\frac{k_{\text{cat}}/K_{m}^{\text{NADH}}\left[\text{NADH}\right]}{1+\left(k_{\text{cat}}/K_{m}^{\text{NADH}}\left[\text{NADH}\right]/k_{\text{cat}}\right)}, \end{array}$$

where *v* stands for the initial velocity, *e* is the enzyme concentration, *K*_*m*_^NADH^ is the Michaelis constant for the enzyme-NADH complex, *k*_cat_ is the turnover number of the enzyme, and *k*_cat_/*K*_*m*_^NADH^  is the enzyme catalytic efficiency.

The reactivity of the CTC towards molecular oxygen was monitored by full reduction of hAIF_∆1-101_ samples with NADH (1.5-fold the concentration of the protein) in 50 mM phosphate buffer, pH 7.4, and following their reoxidation in a Cary 100 spectrophotometer (*Agilent*, Santa Clara, CA). Absorption spectra were recorded at 25 °C until full oxidation of the flavin cofactor was achieved. For each time, the percent remaining of CTC versus reoxidation by molecular oxygen was estimated as Δ*A*_*t*_/Δ*A*_max_, where Δ*A*_max_ is the difference between the minimum and the maximum absorbance at 700 nm, and Δ*A*_*t*_ is the difference of each value at 700nm minus the minimum absorbance at 700 nm. The CTC half-life is the time at which 50% of CTC still remains.

A SX18.MV stopped-flow spectrophotometer (*Applied Photophysics Ltd*., Surrey, UK), interfaced with the ProData-SX software and a photodiode array detector, was used to investigate the fast kinetic reduction of the hAIF variants by the NADH coenzyme. Samples of ~10 *μ*M hAIF_∆1-101ox_ were mixed with increasing concentrations of NADH (0.03-10 mM) under aerobic conditions in 50 mM potassium phosphate, pH 7.4, at 25 °C. The enzyme and NADH concentrations are the final ones obtained after mixing equal volumes of substrate and enzyme. Observed rate constants for the hydride transfer (HT) event (*k*_obs_) were calculated by global analysis and numerical integration methods (simultaneously using all spectral data in the 400-800 nm region along time evolution). A single-step model (A→B) best fitted to describe the overall reaction at all NADH concentrations assayed. Averaged *k*_obs_ values at each NADH concentration were then fitted to the equation that describes the formation of an enzyme:substrate complex prior to the HT event:

(3) $$\begin{array}{c} k_{\text{obs}}=\frac{k_{\text{HT}}\text{NADH}}{K_{d}^{\text{NADH}}+\text{NADH}}+k_{\text{rev}}, \end{array}$$

where *k*_HT_ is the limiting rate constant for HT from the pyridine nucleotide coenzyme to the FAD cofactor of hAIF, *K*_*d*_^NADH^ is the dissociation constant of the transient hAIF_∆1-101ox_:NADH complex, and *k*_rev_ is the reaction constant for a potential overall reverse process.

Stopped-flow spectrophotometry was also used to evaluate the rate constants of CTC formation when mixing photoreduced hAIF_∆1-101_ (hAIF_∆1-101phrd_) with increasing concentrations of NAD^+^ (0.125-5 mM) under anaerobic conditions. hAIF_∆1-101phrd_ samples were obtained by photoreduction in the presence of 5 *μ*M methyl viologen, 3 *μ*M 5-deazariboflavin, and 20 mM EDTA. The assays were performed at 25 °C in 50 mM potassium phosphate, pH 7.4, under anaerobic conditions (obtained by several cycles of vacuum application and bubbling with O_2_ free argon). Data were global fitted to a single step model (A→B), and *k*_obs_ were determined at the different NAD^+^ concentrations assayed. These values were then fitted to the equation that describes the formation of a transient hAIF_∆1-101phrd_:NAD^+^ complex prior to the CTC stabilization:

(4) $$\begin{array}{c} k_{\text{obs}}=\frac{k_{\text{CTC}}\left[{\text{NAD}}^{+}\right]}{K_{d}^{\text{NAD}+}+\left[{\text{NAD}}^{+}\right]}, \end{array}$$

in which *k*_CTC_ is the limiting rate constant for the rearrangement of the encounter complex to form the CTC, and *K*_d_^NAD+^ is the dissociation constant for the mentioned transient encounter complex.

### 2.7. Isothermal Titration Calorimetry (ITC)

ITC assays were carried out using an Auto-iTC200 (*MicroCal*, *Malvern-Panalytical*, Malvern, UK) thermostated at 25 °C. Typically, 10-20 *μ*M protein partner and dsDNA samples—prepared as described below—were used to titrate ~10 *μ*M hAIF_∆1-101_ variants. All solutions were degassed at 15°C for 1 min before each assay. A sequence of 2 *μ*L injections of titrant solution every 150 s was programmed, and the stirring speed was set to 750 rpm. The association constant (*K*_*a*_), the enthalpy of binding (Δ*H*), and the binding stoichiometry (*N*) were estimated through nonlinear least-squares regression of the experimental data employing a single-ligand binding site model implemented in Origin 7.0 (*OriginLab*, Northampton, MA). The dissociation constant (*K*_*d*_), the free energy change (Δ*G*), and the entropy change (Δ*S*) were obtained from basic thermodynamic relationships.

Since hAIF binds DNA unspecifically, a 0.5 mM dsDNA sample was prepared from 1 mM solutions of HPLC-purified forward and a reverse complementary 15-bp oligonucleotides (5′- GGT TAG TTA TGC GCG -3′; randomly designed) synthetized by Integrated DNA Technologies. The pair of oligonucleotides was mixed at an equimolar ratio and annealed by heating 1 min at 99°C and performing a 3 h temperature scanning from 95 to 25°C, decreasing 1°C each 3 min. 0.5 mM dsDNA stock solutions were obtained.

### 2.8. Generation of Structural Models

Models containing the missing C-loop residues (546–558 and 518-559, respectively, for crystal structures of WT hAIF_*Δ*1-101ox_ and hAIF_*Δ*1-101rd_:NAD^+^ states), as well as W196A, W196L, and W196Y mutations, were built using as templates, the coordinates of WT hAIF_*Δ*1-101ox_ (PDB 4BV6) and hAIF_*Δ*1-101rd_:NAD^+^ (PDB 4BUR) and the Swiss-Model server [7, 10, 24]. Routines for minimization and molecular dynamics (MD) simulations followed previous reported protocols [7] and are summarized in the supplementary materials. Improvements include using a time step of 2 fs and performing five replicas of 10 ns MD production for each model structure.

### 2.9. Data Analysis

Data were fit and shown using SigmaPlot (*Systat. Software Inc. Richmond*, CA, USA), Origin 7.0 (*OriginLab Corporation*, Northampton, MA), and Pro-K (*Applied Photophysics Ltd.*, Surrey, UK). VMD [25] and PyMol [26] were used to analyze and visualize structural data, as well as to produce structural figures.

## 3. Results and Discussion

### 3.1. Mutations at W196 Residue Hardly Impacts the Overall hAIF_∆1-101_ Core Conformational Properties in Oxidized and NADH-Reduced States

The three W196 variants here studied were purified to homogeneity as holoproteins after their expression in *E.coli* as described previously for the WT protein [10]. Their UV-visible absorption spectra showed the characteristic bands I and II of the flavin at 451 and 380 nm, respectively, a shoulder at 476 nm, and *A*_280_/*A*_451_ ratio ≈11, indicating that, similarly to the WT protein, the cofactor was in the oxidized state and correctly incorporated to the protein (Figure S2A). Only W196A showed a distorted shape for band II and lower *A*_451_/*A*_380_ ratio reflecting some differences in the environment of its flavin ring.

The W196 variants also had similar far-UV CD spectra to the WT protein, with minima at ~222 and ~208 nm indicative of high *α*-helix content (Figure S2B). Reduction of the FAD cofactor by NADH produced the decrease in relative intensity of minima at 208 nm for all mutants (Figure S2C), as previously reported for the WT protein [7]. This suggests similar overall conformations in the CTCs. The near-UV/Vis CD spectra of the variants showed the WT characteristic maxima (~300 nm and ~365 nm) and minima (~453 and ~477 nm) (Figure S2D). Finally, changes observed upon incubation with NADH were also consistent with FAD reduction (lack of near-UV CD signal at 300 nm and in the 350-500 nm range) and CTC stabilization (new minima at ~405 nm and broad bands at ~600 nm) in all variants (Figure S2E) [7].

Since the crystal structure is only available for W196A_ox_, we built structural models containing the W196 mutations, as well as the missed C-loop residues in the WT X-ray structures, to further evaluate the impact of mutations on the conformation of hAIF_∆1-101ox_ and its CTC [7, 10]. Models for oxidized variants, including W196A_ox_, were built using the WT_ox_ crystal structure as a template to better evaluate the effect of each mutation on native structures, thus preventing the other variant's models from being “forced” to behave as W196A_ox_. After 10 ns MD relaxation, only small fluctuations within each simulated system were detected for averaged values of energy, radius of gyration, RMSD, and solvent accessible surface (SAS) of ligands, as well as for the main interactions coupling the FAD cofactor and NADH coenzyme to the protein (Figures S3A and S4). These observations contrast with those obtained when similarly evaluating the pathogenic deletion of residue R201 situated together with W196 in the *β*-hairpin and also contributing to C-loop linking [7]. This clinical *Δ*R201 variant rapidly breaks the network linking the FAD cofactor, the *β*-hairpin itself, the active site residues, the central *β*-strand, and the C-loop during the MD production [7]. Altogether, experimental and modelling evidences indicate that substitutions at W196 retain the WT hAIF_∆1-101_ architecture at the active site and the protein core, in both the oxidized and CTC states. In agreement, W196A_ox_ was even able to crystallize [11].

### 3.2. W196 Side Chain Modulates the Monomer-Dimer Equilibrium in hAIF_*Δ*1−101_

**Figure 2:**
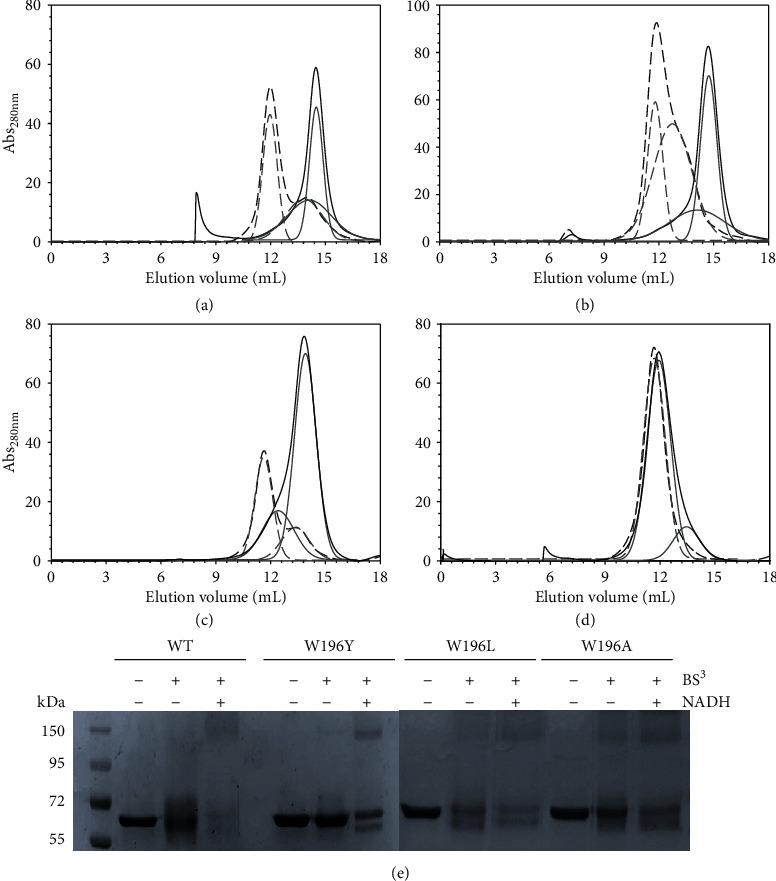
Effect of W196 replacement on the hAIF_*Δ*1-101_ ability to stabilize dimers. Elution profile of (a) WT, (b) W196Y, (c) W196L, and (d) W196A on a Sephadex S-200 column at 6°C. The assays were performed in absence and presence of a 10-fold excess of NADH, and profiles are, respectively, shown in black continuous and dashed lines. The respective different populations assigned by Gaussian analysis are depicted in grey lines. (e) Chemical cross-linking of hAIF_*Δ*1-101_ samples (~3 *μ*M proteins) with a 100-fold excess of the BS^3^ cross-linker in the absence and presence of NADH (300 *μ*M). After 45 minutes of incubation, the reactions were stopped by the addition of bromophenol sample buffer and resolved by 12% SDS-PAGE.

Gel filtration chromatography was used to study the impact of mutations on the ability of hAIF_*Δ*1−101_ to undergo NADH-linked dimerization. While, similarly to the WT_ox_ protein (Figure 2(a)) [10], the W196Y_ox_ mutant eluted as a monomer of apparent molecular weight (^app^MW) ~45-58 kDa (Figure 2(b)), the W196L_ox_ and W196A_ox_ variants eluted as considerably broad peaks with lower exclusion volumes. Peak deconvolution suggested two populations with ^app^MW of 63 and 115 kDa for W196L_ox_ and 75 and 138 kDa for W196A_ox_ (Figures 2(c) and 2(d), respectively), indicating less compact monomeric conformations and/or a quick monomer-dimer exchange. Upon incubation with NADH, the W196Y and W196L variants eluted mainly as a new peak of lower exclusion volume (~145-155 kDa) (Figures 2(b) and 2(c)) that was previously related to the CTC dimer in the WT protein (Figure 2(a)). Finally, the elution peak for W196A in the presence of NADH, when compared to W196A_ox_, also gets narrower and slightly displaced towards the WT CTC dimer elution volume (Figure 2(d)).

Chemical cross-linking with BS^3^—able to covalently conjugate hAIF dimers but not monomers—followed by assessment of species by SDS-PAGE (Figure 2(e)), was then used to evaluate whether the observed chromatographic changes might relate to W196 mutations influencing the compactness of protein conformation and/or the CTC dimer lifetime. Upon incubation with BS^3^, all oxidized mutants exhibited the band of ∼55 kDa corresponding to the hAIF_*Δ*1−101ox_ monomer, although it was in general more diffuse than in the cross-linker absence. When variants were preincubated with both NADH and BS^3^, an additional broad band of ~170 kDa was detected. In WT hAIF_*Δ*1−101_, this band is related to the protein ability to undergo dimerization in the CTC state upon NADH binding and flavin reduction [10]. Noticeably, this band, indicative of dimer stabilization, was also observed for W196A_ox_ (in the absence of the coenzyme), in agreement with the exclusion chromatography data obtained for this variant (Figure 2(d)) and with its reported dimeric crystal structure [11]. These data confirm that all W196 variants are able to dimerize upon NADH reduction, but also show that the mutations modulate the CTC dimer stability. They also suggest conformational changes that favor the displacement of the monomer-dimer equilibrium towards the dimer in the oxidized state, particularly in W196A_ox_.

### 3.3. W196 Highly Contributes to Modulate the Low Efficiency of hAIF_∆1-101_ as NADH Oxidase

**Figure 3:**
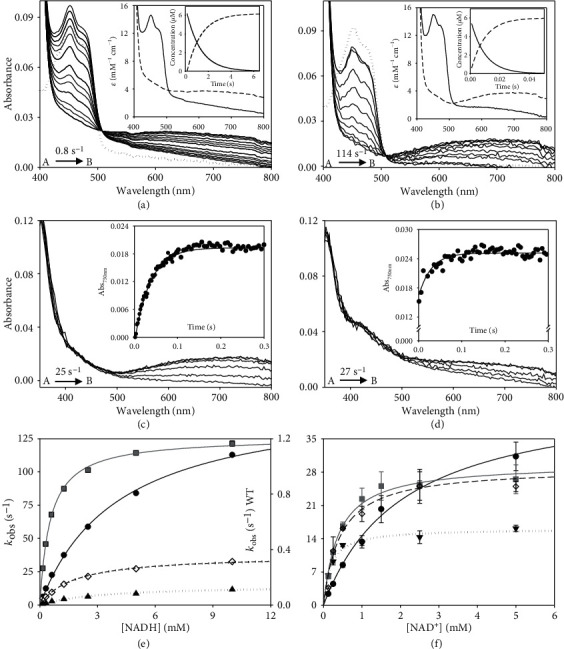
Kinetic characterization of W196 hAIF_*Δ*1-101_ variants. Spectral evolution of the reduction of (a) WT (~10 *μ*M) and (b) W196A variant (~ 10 *μ*M) when mixed with NADH (2 and 5 mM for WT and W196A, respectively). Spectra for the reduction of WT_ox_ are shown at 0.15, 1.05, 2.1, 4.2, 6, 10.05, 12.45, 20.1, 30, 40.05, and 50.1 s after mixing and those for W196A at 0.005, 0.05, 0.1, 0.15, 0.2, 0.25, 0.3, 0.45, 0.5, and 0.55 s. Dotted lines correspond to the spectra of oxidized enzymes before mixing with the coenzyme. The corresponding insets show the absorbance spectra for the intermediate species obtained by fitting the spectral evolution to a single step model (A→B) and the evolution of the concentration of each species. Kinetics of CTC formation upon mixing of the hAIF_∆1-101phrd_ forms of (c) WT and (d) its W196A variant with NAD^+^ (5 mM) under anaerobic conditions. Spectral evolution for CTC formation of WT at 0.001, 0.005, 0.01, 0.015, 0.02, 0.07, 0.1, 0.15, 0.2, 0.33, 0.4, and 0.5 s after mixing and those for W196A at 0.001, 0.002, 0.005, 0.006, 0.007, 0.008, 0.009, 0.01, 0.03, 0.05, 0.07, 0.09, 0.1, 0.3, and 0.5 s. The corresponding insets show the absorption evolution at 750 nm (black circle) and the fits (continuous line) at this wavelength after globally fitting evolution at a single step model (A→B). (e) Dependence of the observed rate constants for flavin reduction for the WT (black circle), W196Y (black triangle), W196L (black diamond), and W196A (grey square) reactions on the NADH concentration. Lines represent the fits of experimental data to equation (3). (f) Dependence of observed rate constants for CTC formation when using WT (black circle), W196Y (black triangle), W196L (black diamond), and W196A (grey square) hAIF_∆1-101phrd_ on the NAD^+^ concentration. Lines represent the fit of experimental data to equation (4). Assays were performed in a stopped-flow spectrophotometer in 50 mM potassium phosphate, pH 7.4, and at 25°C (*n* = 3, mean ± SD).

**Scheme 1:**
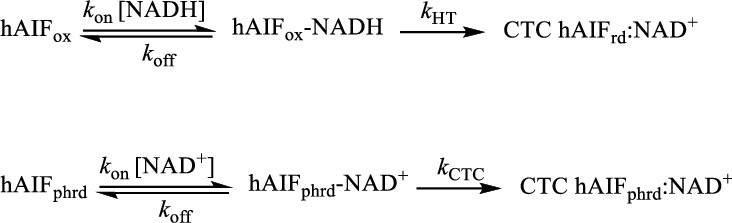


Under physiological conditions, hAIF exhibits a NADH oxidase activity that can be *in vitro* monitored using the steady-state DCPIP-dependent diaphorase reaction. When evaluated in this way, all W196 variants showed higher turnover rates than the WT protein (~3-fold increase for W196Y and W196L and ~5-fold for W196A) (Table 1). Regarding *K*_m_^NADH^, the W196Y variant value was similar to that for the WT, while the W196L and W196A variants showed a significant decrease (~3- and 10-fold, respectively). Thus, W196Y, W196L, and W196A variants were ~3, ~8, and~45 times more efficient oxidizing NADH than the WT protein. Nonetheless, despite these W196 variants are more efficient as oxidoreductases than WT hAIF_*Δ*1−101_, they were unable to oxidize NADH when using molecular oxygen as electron acceptor, analogously to the WT protein [10]. In the light of these results, we studied the impact of the W196 mutations on the HT reaction from NADH to the FAD cofactor of hAIF_∆1-101_ by using stopped-flow transient kinetics. The kinetic traces recorded for all variants at different NADH concentrations indicated an essentially irreversible two-electron reduction of the FAD cofactor and the concomitant formation of a long wavelength broad band related to the stabilization of the hAIF_∆1-101rd_:NAD^+^ CTC species (Figure 3 and S5). The intensity of this CTC band (area in the 510−800 nm region minus that of the free protein) for W196A and W196Y variants was in the range of that observed for the WT [10], suggesting similar percentage of CTC stabilization. However, the lower intensity of W196L CTC band (∼76%) indicates either different charge distribution between coenzyme and FAD rings in the CTC (suggestive of different CTC geometry) or reduction of the amount of the CTC stabilized. In all cases, global analyses of the spectral range time evolutions best fitted to a one-step model (A→B). Thus, the observed processes appeared including the fast formation of the transient hAIF_∆1-101_:NADH reactive complex followed by the HT reaction and the CTC formation (Scheme 1). As a consequence, the conformational switches in *β*-hairpin and C-loop induce protein dimerization in W196 variants, with the potential exception of W196A that presumably might be mostly a dimer with the C-loop already released in the oxidized state [11]. The *k*_obs_ values obtained showed hyperbolic dependence on NADH concentration for all variants, allowing *k*_HT_ and *K*_*d*_^NADH^ determination upon fitting to the equation (4) (Figure 3(e) and Table 1). All variants showed faster HT rate constants and higher affinity for the NADH substrate than the WT protein (up to ~29- and 5-fold, respectively, for W196A). Consequently, W196Y, W196L, and W196A were ~12-, ~24-, and up to ~153-fold more efficient than the WT enzyme as hydride acceptors from NADH. Noticeably, and, contrary to that described for the WT protein, all these W196 variants showed *k*_HT_ values higher than their turnover rates, suggesting that for them, the HT reaction is not the limiting step during catalysis. Therefore, the W196 side chain highly contributes to modulate the properties of hAIF_∆1-101_ as a nonefficient NADH oxidase.

**Figure 4:**
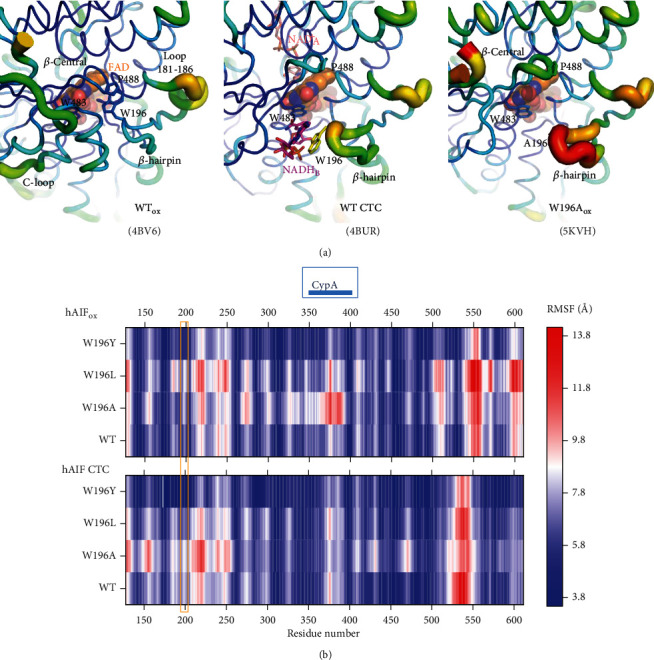
Impact of mutations on hAIF_*Δ*1-101_ flexibility. (a) B-factor putty representation of crystal structures of WT hAIF_*Δ*1-101ox_ (4BV6), WT CTC (4BUR), and W196A hAIF_*Δ*1-101ox_ (5KVH) focusing in the environment of the *β*-hairpin containing residue 196 and the central *β*-strand. Orange to red colors and a wider tube indicate regions with higher B-factors, whereas shades of blue and a narrower tube indicate regions with lower B-factors. The FAD cofactor is shown as sphere representation with carbons in orange, while, when present, the NAD^+^_A_ and NADH_B_ are shown as sticks with carbons in salmon and pink, respectively. (b) Heat map representation of the average C*α* root mean square fluctuation (RMSF) of five replicates for 10 ns MD simulations of the different W196 variants in both the hAIF_*Δ*1-101ox_ and CTC states. The region corresponding to the *β*-hairpin is highlighted with an orange square and that predicted for interaction of CypA is indicated on the top of the panel.

**Figure 5:**
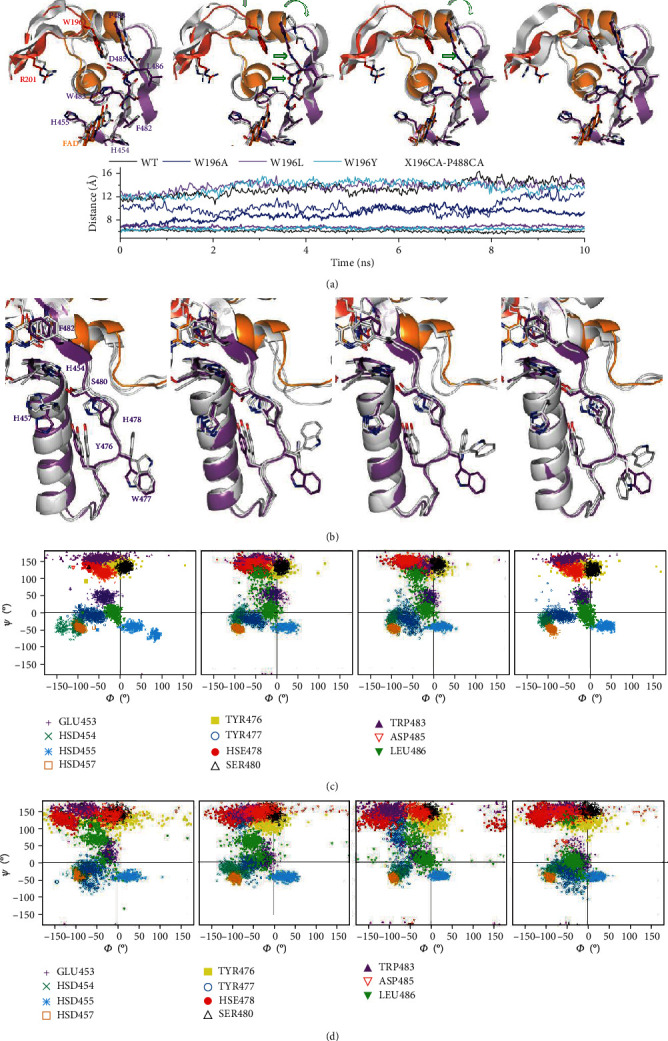
Impact of W196 hAIF_*Δ*1-101_ mutations on the dynamics of the *β*-hairpin and its environment. Representative conformations of the (a) *β*-hairpin and the central *β*-strand and the (b) His-rich helix and the loop connecting it to the central *β*-strand in final MD structures of selected replicates for each W196_ox_ variant. MD final replicates are shown in grey in each panel. All panels compare to the crystallographic WT_ox_ structure (4BV6) that shows the *β*-hairpin in red, the C-loop in orange, and the rest of the protein in purple. Open green arrows indicate relevant displacements of structural elements relative to the crystallographic WT_ox_ structure. The lower panel in (a) shows the time evolution of the distances between C*α*s of the residue at position 196 in the *β*-hairpin and P488 in the *β*-sheet. For each variant, data show averaged values for the 5 MD replicates run for each model in the hAIF_∆1-101ox_ (bold lines) and CTC (line) states. Ramachandran plots of the distribution of key main chain *Φ*/*ψ* conformational along the MD of selected replicates for each (c) oxidized and (d) reduced W196 variant.

Structurally, W196 does not form part of the protein redox active site itself. However, W196 side chain stacks to P488 at the edge of the central *β*-strand, contributing to situate the *β*-hairpin and the C-loop forming a cavity at the bottom of which sits W483—a residue that flanks the pyrimidine ring of FAD—(Figure 1(b)). W196A mutation increases W483 solvent accessibility and C-loop and *β*-hairpin flexibility, favoring their displacement from the WT_ox_ positions (Figures 1(c)–1(e) and 4). Noticeably, we observed the *β*-hairpin displacement from P488 as well as the central *β*-strand retraction from the beginning of our W196A_ox_ model MD trajectories (starting from WT_ox_ structure) (Figure 5(a)). However, the Y196 and L196 side chains contribute to maintain *β*-hairpin position in the W196Y_ox_ and W196L_ox_ trajectories, and retraction of the central *β*-strand is hardly deduced for W196L_ox_ (Figure 5(a)). Trajectories also show a larger increase in the SAS of the *β*-hairpin of W196A_ox_ relative to the other two variants and the WT (Figure S3B). Thus, MD simulations predict an increase in distances between W483 and atomic positions at the active site of the oxidized variants (Figure S4A). Such changes in W483 solvent accessibility and active site compression must impact substrate affinity and coupling into a competent complex for HT, as well as the FAD midpoint reduction potential and/or electronic distribution—as was previously reported for the murine W196A variant (70 mV higher redox potential than those for the WT protein)—[9]. In agreement, kinetic parameters show W196A as the variant differing more from the WT behavior regarding efficiency for both HT and NADH binding, followed—by far—by W196L and being the aromatic substitution the one producing a milder effect. Dynamics of active site in CTCs show higher flexibility regarding oxidized state (Figure S4A-B), but WT CTC keeps its characteristic parallel ionic pair through stacking of the NAD^+^ nicotinamide and FADH^−^ isoalloxazine reacting rings (Figure S4D). In such arrangement, the angle formed between the C4n hydride donor of the nicotinamide of the coenzyme, the hydride to be transferred and the N5 acceptor atom of the FAD isolloxazine ring (C4n-hydride-N5) appears far from collinearity, involving a large free energy penalty for geometric deformations and partial loss of the *π* stacking interaction to achieve the transition state [27, 28]. Therefore, despite the C4n-N5 distance being compatible with HT, the hydride shift can be quite inefficient. This seems to be the situation for HT in WT, showing inefficient *k*_cat_ and *k*_HT_ values for NADH oxidation. Noticeably, MD simulations for the W196 CTC variants suggest the distortion of the FADH^−^:NAD^+^ pair and the pulling apart of C4n and N5 reacting atoms (Figure S4C). This effect is observed in W196A and W196L CTCs and to a lower extent in W196Y CTC, in agreement with improvement in their *k*_HT_ parameters. Therefore, the size and aromaticity of the side chain of W196 in hAIF_∆1-101_ are key to fix the *β*-hairpin position and, despite not forming part of the active site itself, set the active site geometry to make it a nonefficient NADH oxidase.

### 3.4. W196 Contributes to Stabilize the CTC State

Changes above detected in *K*_*m*_^NADH^ and *K*_*d*_^NADH^ envisage an important impact of mutations at W196 on the association/dissociation equilibria of NADH and NAD^+^ to hAIF_∆1-101_. Therefore, we proceeded to evaluate transient rate constants for CTC formation as well as stability of the CTCs once formed. To determine rate constants for CTC formation, we mixed hAIF_∆1-101phrd_ samples with different concentrations of NAD^+^ in the stopped-flow equipment (Figures 3(c) and 3(d), and Figure S5). For all W196 variants, kinetic traces showed CTC formation following an essentially irreversible two-species process with *k*_obs_ values showing hyperbolic dependence on NAD^+^ concentration (Figure 3(f)). This allows to determine *K*_*d*_^NAD+^ as well as the limiting kinetic constant related to the establishment of the nicotinamide:isoalloxazine electronic exchange within the CTC species (*k*_CTC_) (Table 1, Scheme 1). Mutations at W196 produce a strong impact in *K*_*d*_^NAD+^, whose value decreases ~5-fold in the Leu and Ala variants and up to ~11-fold in the Tyr one. This indicates stronger affinity for NAD^+^ in the case of the reduced mutants regarding the WT counterpart. On the contrary, the introduced mutations slightly decrease *k*_CTC_ values (up to less than 3-fold for W196Y). Therefore, W196 replacement induces stronger affinity of hAIF_∆1-101phrd_ for NAD^+^ relative to the oxidized state, but hardly influences the kinetic evolution of the initial transient interacting complex to achieve the final CTC conformation. These data indicate that replacements at W196 kinetically favor CTC formation by increasing the hAIF_∆1-101phrd_ affinity for NAD^+^. Noticeably, comparing *K*_*d*_^NADH^ and *K*_d_^NAD+^ values (Table 1), all W196 variants increase the thermodynamic preference for the binding of NAD^+^ over that for NADH regarding the WT. This agrees with the high impact of mutations on *k*_HT_ becoming considerably milder in *k*_cat_ values, where release of the NAD^+^ product will limit the overall oxidoreductase activity.

**Figure 6:**
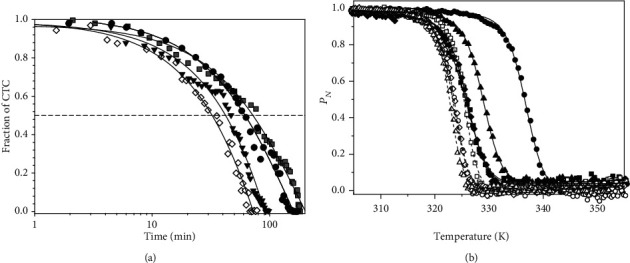
Effect of W196 replacements on the CTC half-life and the thermal stability of hAIF_*Δ*1-101_. (a) Reactivity of the CTC towards O_2_ in W196 hAIF_*Δ*1-101_ variants. hAIF_∆1-101rd:_NAD^+^ CTC decay was monitored at 750 nm and 25°C in air saturated 50 mM potassium phosphate at pH 7.4. CTC hAIF_∆1-101rd:_NAD^+^ samples were obtained by mixing hAIF_*Δ*1-101ox_ variants with NADH (0.7-fold the enzyme concentration). The traces for WT (black circle), W196Y (black triangle), W196L (black diamond), and W196A (grey square) hAIF_*Δ*1-101_ are shown normalized from 1 to 0 as fraction of CTC remaining along the time. The solid line represents the fit of the traces to a single-exponential decay process to determine CTC half-life. (b) The thermal stability for flavin release (*T*_*m*_^FAD^) of hAIF_∆1-101_ variants. Curves for FAD thermal release in oxidized variants (closed symbols) and its CTC state (open symbols), as monitored by increase in FAD fluorescence emission upon protein denaturation. The WT, W196A, W196L, and W196Y hAIF_∆1-101_ are in black circle, black square, black diamond, and black triangle, respectively. The curves are roughly normalized to the change in fluorescence signal of the FAD bound fraction (*P*_*N*_, from 1 to 0), with their fits to a two-transition unfolding model (continuous and dashed lines for oxidized and reduced states, respectively). Decrease in FAD bound fraction was experimentally followed by the increase in its fluorescence upon release from the holoprotein along a 20 to 85°C temperature ramp. Data were obtained in 50 mM potassium phosphate at pH 7.4 and at a final ionic strength of 150 mM. Protein concentration was ~2 *μ*M. The CTC forms were obtained by premixing hAIF_∆1-101ox_ and NADH at a 1 : 100 ratio.

We then evaluated the mutational effect on reactivity towards O_2_ for the CTC formed upon NADH oxidation. As shown in Figure 6(a), the W196A CTC was highly stable versus reoxidation by O_2_, similarly to the WT CTC, (half-lives of 62 and 72 min, respectively), while CTC lifetimes were slightly shorter for W196Y and W196L (half-lives of 42 and 32 min, respectively). Therefore, Tyr and Leu replacements favor O_2_ access to the FAD cofactor in the CTC. Altogether, these observations point to W196 and the *β*-hairpin conformation also contributing to the strength of NADH/NAD^+^ binding to the hAIF active site.

### 3.5. W196 Mutations Reduce Thermostability of hAIF_*Δ*1−101ox_, but Not of the CTCs

Fluorescence emission curves reflecting FAD release upon protein thermal denaturation were then obtained to evaluate the effect of W196 substitution on FAD binding and hAIF_*Δ*1−101_ stability (Figure 6(b) and Table 2). All W196_ox_ variants were less stable than WT_ox_, with *T*_*m*_^FAD^ dramatically decreasing between ~8 and ~11 °C. Formation of the WT CTC upon NADH mixing has an even more negative impact in WT hAIF_*Δ*1−101_ stability, decreasing *T*_*m*_^FAD^ for the CTC (*T*_*m* CTC_^FAD^) by ~13 °C relative to *T*_*m*_^FAD^ [7]. This destabilizing effect was related to release of the regulatory C-loop in the hAIF_*Δ*1−101_ apoptotic domain promoting looser tertiary structure contacts at the active site fostering cofactor dissociation [7]. Interestingly, all reduced CTC variants showed similar *T*_*m* CTC_^FAD^ to the WT CTC. This reflects that CTC formation induces a considerably lower destabilizing effect in W196L and W196Y relative to their oxidized states than in the WT case (*T*_*m* CTC_^FAD^ ~2 and ~6 °C lower than their *T*_*m*_^FAD^, respectively), while no destabilization is produced at all for W196A. These data suggest that the decrease in compactness of the hAIF_*Δ*1−101ox_ active site depends on W196 mutation. In agreement, structural predictions indicate that W196A_ox_ has a larger propensity for *β*-hairpin release, increasing of *β*-hairpin SAS as well as conformational rearrangement of the central *β*-strand and the loop connecting it to the His-rich helix (Figure 5 and Figure S3B). Thus, W196A_ox_ backbone dynamics at these regions envisages a behavior more similar to the CTC than to WT_ox_, while an intermediate situation appears for W196L_ox_ and W196Y_ox_ (Figures 5(c) and 5(d)). On this side, the very low impact of mutations on *T*_*m* CTC_^FAD^ agrees with conformational rearrangements already produced upon CTC formation, having the W196 side chain less relevance in contributing to the active site compactness. Finally, since, according to the WT CTC crystal structure, the W196 side chain contributes to stack the adenine moiety of NADH_B_ (Figure 1(e)), such lack of effect is suggestive of NADH_B_ binding being considerably weaker than that of NADH_A_. This is clearly supported by the larger SAS for NADH_B_ over NADH_A_ in the MD simulations, which is in addition independent of the W196 variant considered (Figure S3A). Therefore, these thermal stability analyses suggest that mutations at W196 decrease the compactness of hAIF_*Δ*1−101ox_. Noticeably, some of the pathogenic mutations located at *β*-hairpin, such as ∆R201, or involved in interaction between the *β*-hairpin and the regulatory C-loop, such as F210S/L, are reported to also show diminished compactness and/or stability [7, 29, 30].

### 3.6. W196 Contributes to Modulate the Conformation of the Interaction Surfaces of hAIF_*Δ*1−101_ with Its Physiological Partners

**Figure 7:**
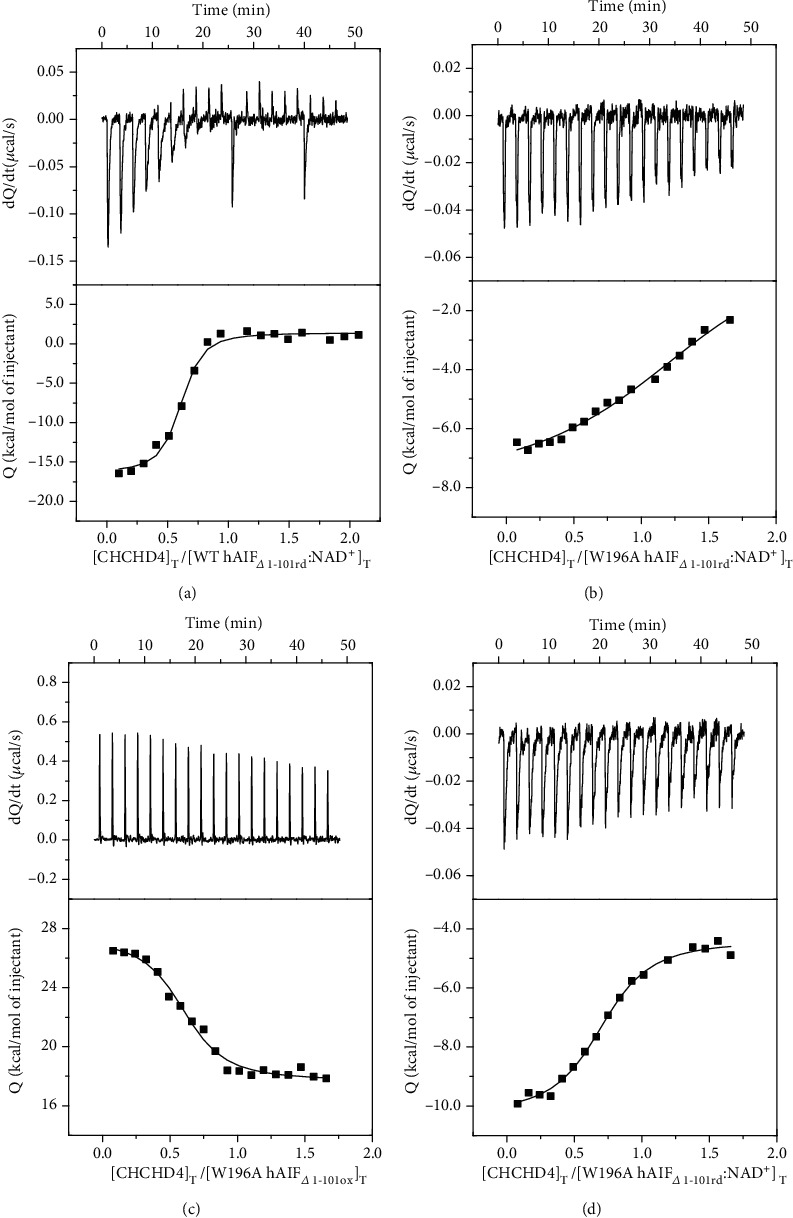
Effect of the W196 replacement in the binding of CHCHD4 to hAIF_*Δ*1-101_. Calorimetric titrations of (a) WT CTC, (b) W196Y CTC, (c) W196A_ox_, and (d) W196A CTC with CHCHD4. The upper plots show the thermograms (thermal power as a function of time), whereas the lower plots show the binding isotherm (normalized heats as a function of the CHCHD4/hAIF molar ratio). Measurements were carried out in 50 mM potassium phosphate, pH 7.4, at 25°C. The CTC forms were obtained by premixing hAIF_∆1-101ox_ and NADH at a 1 : 100 ratio. The binding parameters were estimated through nonlinear least-squares regression applying a single-ligand binding model (continuous lines in binding isotherms).

AIF plays a key role in cell death and life through its interaction with nucleotides, but also with DNA and a broad number of proteins. We selected some representative ligands to evaluate the mutational effect of W196 on the hAIF interaction network: CHCHD4 as a mitochondrial partner key in OXPHOS and energy homeostasis, as well as the nuclease CypA and DNA as nuclear partners for chromatinolysis and PCD. We determined the binding parameters that describe the formation of binary complexes using ITC (Table 3, Figure 7 and S7). Due to the observed W196L tendency to denature during ITC assays, these studies were restricted to WT, W196A, and W196Y variants. For all ligands, the binding isotherms adequately fitted to a model of a single binding site with *K*_*d*_ in the micromolar range.

A number of evidences place CHCHD4 in the pathway linking hAIF to the biogenesis of mitochondrial complexes by facilitating the mitochondrial import of CHCHD4 and its proper localization in IMS [12]. Nonetheless, heat exchange was not observed when titrating WT_ox_ and W196Y_ox_ with CHCHD4, resulting in binding thermogram characteristic of noninteracting systems (not shown). In contrast, the titration of W196A_ox_ with CHCHD4 revealed a strong binding of CHCHD4 to W196A_ox_ (*K*_*d*_~0.4 *μ*M). Binding was entropically driven with the enthalpic contribution being unfavorable (Figure 7(c) and S8A) and suggesting that nonspecific forces contribute to this interaction. When we analyzed the interaction of all CTC variants—obtained by preincubation with NADH—with CHCHD4 (Figure 7 and S8A), binding was detected, in agreement with previous studies that described such interaction as NADH dependent [13]. The WT CTC:CHCHD4 interaction was driven by a large favorable enthalpic contribution indicative of specific binding, while the entropic contribution was unfavorable. This suggests a structurally more organized complex than its separated protein components. W196Y CTC and W196A CTC showed a thermodynamic profile similar to that of WT CTC, but the mutations decreased the magnitude of the favorable enthalpic contribution to the binding and made the entropic term considerably less and slightly more unfavorable, respectively. Regarding CHCHD4 affinity, no significant effect was produced by the alanine substitution in both redox states relative to WT CTC, but the W196Y CTC significantly decreased it (*K*_*d*_~10-fold higher). Structurally, W196A_ox_ in solution appears to be mainly a dimer with a disordered C-loop [11], sharing these features with WT CTC. Thus, our data might support an essential role of the arrangement of either the dimer conformation or the site exposed by C-loop displacement or even the C-loop itself in the interaction with CHCHD4. Such observations agree with deletion mutants and pathogenic point mutations at the NADH domain of hAIF that compromise NADH oxidase activity and CTC structural rearrangements, consequently affecting CHCHD4 binding and resulting in mitochondriopathy phenotypes [13, 31]. However, the W196A_ox_ enthalpic/entropic thermodynamic binding profile for CHCHD4 indicates nonspecific interactions, while those for CTCs suggest specific organized interactions (Figure S8, Table 3). A defined interacting region for CHCHD4 has not been identified yet in hAIF, suggesting a relevant role for its entire ternary organization. On the contrary, CHCHD4 binds through a short N-terminal 27-amino-acid-long fragment, and its redox state appears irrelevant for the interaction. Moreover, this N-terminus is unstructured when CHCHD4 is free [32], but appears to get a defined and organized structure when interacting with the CTC [13]. This agrees with our data that clearly indicates a specific CHCHD4 binding to WT CTC. On the contrary, the thermodynamic parameters here reported for CHCHD4 binding to W196A_ox_ suggest that, despite the conformational changes produced by the mutation, CHCHD4 recognition is allowed and the complex formed will be far from the specific interaction presumably produced with WT CTC. Therefore, structural NADH-dependent changes in AIF must play a key role in the binding of CHCHD4. This is further supported by the Tyr and Leu replacements in W196 also having a strong impact in the specificity and organization of this interaction. Altogether, these observations suggest that (i) the competent interaction of hAIF with CHCHD4 relies on the adequate CTC architecture to favor the assembly of potentially disordered regions from both proteins to achieve a specific conformation. (ii) W196 contributes to provide such CTC architecture, by potentially regulating the proper *β*-hairpin configuration that is key for the structural transition of hAIF and its role in mitochondrial homeostasis. In agreement, a nonproductive interaction between hAIF and CHCHD4 has been suggested for the clinical mutation F210L that results in abnormal assembly of mitochondrial complexes I and III [30].

Parameters for the titration of WT_ox_ with CypA indicated the enthalpically driven formation of a binary complex with unfavorable entropic binding contribution (Table 3, Figures S7A and S8B). This agrees with the large number of electrostatic contacts reported for the CypA interaction with the AIF synthetic peptide (370-394) [33–35], as well as with the complex adopting a large complementarity of the two proteins. Noticeably, the enthalpic contribution to the CypA binding turned into unfavorable for W196Y_ox_ and W196A_ox_ (Table 3, Figures S7B-C and S8B). However, this is compensated by a change in the sign of the binding entropic contribution that became highly favorable and made the mutational effect being insignificant regarding overall affinity (enthalpy-entropy compensation). Nevertheless, the mutational switch in thermodynamic contributions to the hAIF:CypA interaction suggests specificity decrease and production of nonnative-like conformations. This is interesting, because W196 and the *β*-hairpin do not share the protein surface with the AIF NADH domain where the 370-394 *β*-strand (binding spot for CypA) is situated (Figures S8E and S9). Nonetheless, replacements at W196 have important effects in hAIF_*Δ*1−101ox_ stability (Table 2), as well as in the conformation of the C-loop, the central *β*-strand, and the dimerization interface located far away from W196. Therefore, conformational changes occurring in the *β*-hairpin might be also transmitted to the CypA binding site identified in hAIF. These conformational changes on one side might hinder charge contacts of the 370-394 *β*-strand to CypA, while on the other they might favor CypA binding to hAIF by C-loop structural transition towards a more organized and favorable conformation as indicated by the entropy becoming favorable. In agreement, reduction of the flexibility of a stapled hAIF (370-394) peptide analogue constructed to stabilize its *β*-strand organization as in hAIF considerably improved its affinity for CypA [35]. Therefore, the higher specificity of WT_ox_ for CypA versus the W196_ox_ variants might derive from its increased conformational rigidity favoring a lock-and-key mechanism of recognition, while increasing protein flexibility turns into an unspecific interaction.

Finally, positive charges clustered along the AIF surface are likely to contribute to DNA binding, but a clear sequence specificity is not expected since AIF recognizes DNA and RNA, as well as a large panel of ribonucleoproteins [36]. Our thermograms for WT_ox_ titration with dsDNA further confirm such lack of specificity, since they show an entropically driven binding with an unfavorable enthalpy contributing to the interaction (Figures S7D and S8C, Table 3). W196A and W196Y mutations hardly impair the hAIF_*Δ*1−101ox_ affinity for DNA in binary complexes (*K*_*d*_ values~4-fold higher than WT). However, although thermodynamic patterns resembled the WT ones, the entropic and enthalpic binding contributions for W196A and W196Y variants were more favorable and less unfavorable, respectively (Figures S7E-F and S8C, Table 3). Structurally, binding of DNA to hAIF_ox_ is proposed to occur through the nucleotide strand wrapping around a positively charged protein crown. This crown appears considerably modulated in shape as well as in accessibility when comparing crystallographic WT_ox_ to W196A_ox_ structure as well as in MD models for all variants (Figure S9), in agreement with the modulation observed in experimental binding parameters.

Altogether, these results show that the W196 side chain influences the enthalpic and entropic contributions to the free energy of hAIF binding in binary complexes with CHCHD4, CypA, and DNA. Whereas, its replacement has in general a negative impact on the enthalpic binding contribution, while improves the entropic one (with the only exception of W196A CTC:CHCHD4 complex). Therefore, W196 contributes to stabilize the conformation of the interaction surfaces of hAIF with CHCHD4, CypA, and DNA.

### 3.7. W196 Contributes to Control the hAIF Conformational Landscape to Adapt to Its Physiological Roles

AIF is a moonlight protein with functions in the mitochondria, cytosol, and nucleus, where it appears to behave as a redox sensor of NAD(H/^+^) cellular levels [9–11]. The cellular redox state (NAD^+^/NADH ratio) may modulate the AIF conformational landscape regarding both overall protein conformation and quaternary organization, which in turn seems to be critical for establishing its biomolecular interaction network. The regulatory C-loop in AIF is a predicted internally disordered region that tends to adopt an organized conformation in the protein oxidized state (Figure 1, Figure S8 and S9), but that is released upon NADH-dependent protein reduction, CTC formation, and protein dimerization. The structural properties of W196A_ox_ suggested that the W196 side chain and the *β*-hairpin coupling to the C-loop are key to modulate the structural transition of hAIF in a cellular context.

In healthy mitochondria, hAIF is present in a monomer-dimer equilibrium—regulated by the cytoplasmic NADH pools—that modulates its participation in respiratory complex assembly by physical interactions with CHCHD4 [10, 12–14]. This AIF switching may be critical for maintaining mitochondrial homeostasis along changes in NAD^+^/NADH ratios in response to diet, diseases such as neurodegenerative disorders, and other processes associated to NAD-consuming enzymes—particularly PARP-1 whose activity is increased during aging due to DNA damage accumulation—[37, 38]. In response to NAD^+^ depletion by hyperstimulation of PARP-1, hAIF is released from the mitochondria to the cytosol, allowing its translocation to the nucleus and promoting parthanatos cell death. PARP-1 binding to AIF has been proved to mediate its release from IM by likely inducing conformational changes in the protein [39]. Curiously, the expression levels of AIF were found to be gradually decreased during development and growth in spiral ganglion neurons involved in auditory neuropathy spectrum disorder, a disease caused by point mutations in hAIF, while increased in the aging-related cell dysfunctions where the hAIF role as apoptosis inducer might be more important [40]. To investigate the different potential roles of AIF during development and aging and their regulatory mechanisms, future studies will be required.

In the last years, a significant number of rare mitochondrial diseases caused by more than 20 point mutations in the *AIFM1* gene have been identified. Some mutations in the cell death domain give rise to phenotypes with progressive disorders from childhood, as the Cowchock syndrome. On their side, mutations affecting the hAIF reductase properties decrease the content of respiratory complexes and produce cell respiration deficiencies, while some of them prevent in addition the correct folding of the protein by decreasing its conformational stability. In this later case, the search of molecular chaperones represents an alternative therapeutic strategy yet poorly explored [7, 31, 41–44]. These mutations produce serious mitochondrial encephalopathies, in many cases with severe processes of neurodegeneration and early death. Noticeably, all characterized pathogenic hAIF mutants show a substantially decrease in CTC lifetime, suggesting that its stability is crucial for mitochondrial homeostasis and human health [18, 45]. Further molecular and cellular studies will be required to determine the impact of these pathogenic mutations on the hAIF intracellular processing and interaction with its physiological partners, as well as their link to their multiple clinical neurodegenerative phenotypes.

## 4. Conclusions

This report provides insights into the role in hAIF of W196 and *β*-hairpin motif in the molecular basis of its cellular activities. Our mutational study shows that, contrary to the pathogenic *Δ*R201 mutation—another residue located in the *β*-hairpin and involved in the interaction with the regulatory C-loop—, changes at W196 residue hardly impact the overall conformational folding of hAIF in oxidized and NADH-reduced states. Nonetheless, W196 is key to stabilize *β*-hairpin motif conformation by contacts that are substantially diminished and impaired in all characterized W196 variants. Moreover, the W196 and the *β*-hairpin motif conformation strongly modulate the redox-linked monomer-dimer structural transition in hAIF. The size and aromaticity of the side chain of W196 is key to (i) maintain the proper *β*-hairpin position that stabilizes and retains the regulatory C-loop in the protein score of oxidized hAIF, favoring protein compactness and stability; (ii) configure the NADH active site making hAIF inefficient for NADH oxidation and trigger the C-loop release to the solvent in the reduced state: critical factors for CTC stability and mitochondrial homeostasis; and (iii) define the interaction surfaces with CHCHD4, CypA, and DNA, by modulating the enthalpic and entropic contributions to the free energy of binding. These features contribute to modulate hAIF monomer-dimer equilibrium in a cellular context, which might be relevant for its proper function as a redox sensor of NAD(H/^+^) levels and for its interaction network.

## Figures and Tables

**Table 1:** Steady-state and pre-steady-state kinetic parameters of WT hAIF_∆1-101_ and its W196 variants.

hAIF	Steady-state			Pre-steady-sate					
	*k*_cat_ (s^−1^)	*K*_*m*_^NADH^ (*μ*M)	*k*_cat_/*K*_*m*_^NADH^ (s^−1^mM^−1^)	*k*_HT_ (s^−1^)	*K*_*d*_^NADH^ (*μ*M)	*k*_HT_/*K*_*d*_^NADH^ (s^−1^mM^−1^)	*k*_CTC_^1^ (s^−1^)	*K*_*d*_^NAD+^ (*μ*M)	*k*_CTC_/*K*_*d*_^NAD+^ (s^−1^mM^−1^)
WT	0.9 ± 0.1	495 ± 170	1.9 ± 0.9	1.5 ± 0.1	4090 ± 300	0.4 ± 0.1	45 ± 2	2080 ± 250	22 ± 3
W196Y	2.7 ± 0.1	505 ± 35	5.3 ± 0.6	12 ± 1	2870 ± 320	4.0 ± 0.8	16 ± 1	183 ± 27	87 ± 14
W196L	2.8 ± 0.1	187 ± 18	15 ± 1.9	36 ± 1	1725 ± 210	21 ± 3	29 ± 2	433 ± 11	67 ± 18
W196A	4.3 ± 0.2	25 ± 4	172 ± 35	126 ± 1	1070 ± 40	117 ± 5.3	30 ± 1	394 ± 50	76 ± 10

Assays were performed at 25°C in 50 mM potassium phosphate, pH 7.4 (*n* = 3, mean ± SD). ^1^Kinetic parameters for CTC formations were obtained with hAIF_∆1-101phrd_ variants.

**Table 2:** Thermal stability for flavin release of WT and W196 hAIF_∆1-101_ variants in oxidized and CTC states.

hAIF	hAIF_∆1-101ox_			CTC hAIF_∆1-101rd:_NAD^+^			
	*T*_*m*_^FAD^ (K)	Δ*H* (kcal/mol)	Δ*T*_*m*_^FAD^ (K)	*T*_*m* CTC_^FAD^ (K)	Δ*H* (kcal/mol)	Δ*T*_*m* CTC_^FAD^ (K)	*T*_*m* CTC_^FAD^ − *T*_*m*_^FAD^ (K)
WT	337 ± 1	128 ± 3	--	324 ± 1	150 ± 4	--	−13 ± 2
W196Y	329 ± 1	123 ± 3	−8 ± 2	323 ± 1	167 ± 4	−1 ± 2	−6 ± 2
W196L	326 ± 1	118 ± 3	−11 ± 2	324 ± 1	159 ± 4	0	−2 ± 2
W196A	326 ± 1	103 ± 3	−11 ± 2	326 ± 1	170 ± 4	2 ± 2	0

Values obtained by fitting fluorescence thermal denaturation curves to a two-state unfolding model. Data obtained in 50 mM potassium phosphate, pH 7.4, at a final ionic strength of 150 mM, from 283.15 to 363.15 K. Protein concentrations was ~2 *μ*M, and NADH concentration was in 100-fold excess (*n* = 3, mean ± SD).

**Table 3:** Thermodynamic parameters for the binary interaction of hAIF_∆1-101_ variants with CHCHD4, CypA, and DNA.

hAIF_∆1-101_ form	Titrating ligand	*K*_*d*_ (*μ*M)	Δ*H* (kcal/mol)	Δ*G* (kcal/mol)	*-T*Δ*S* (kcal/mol)
WT_ox_ and W196Y_ox_	CHCHD4	No detected binding			
W196A_ox_	CHCHD4	0.4	9.6	-8.4	-18.0
WT CTC	CHCHD4	0.2	-20.1	-8.7	11.4
W196Y CTC	CHCHD4	2.1	-8.2	-7.5	0.7
W196A CTC	CHCHD4	0.5	-5.9	-8.3	14.2
WT_ox_	CypA	2.7	-24.5	-7.3	17.2
W196Y_ox_	CypA	1.0	17.0	-7.9	-24.9
W196A_ox_	CypA	1.8	27.3	-7.6	-34.9
WT_ox_	dsDNA	1.6	4.8	-7.6	-12.4
W196Y_ox_	dsDNA	6.9	19.2	-6.8	-26.0
W196A_ox_	dsDNA	5.8	15.0	-6.9	-22.0

Values obtained from ITC assays at 25°C in 50 mM potassium phosphate, pH 7.4. *n* is the calculated biding stoichiometry. The thermodynamic parameters were calculated by *K*_*d*_ = (*K*_*a*_)^−1^, Δ*G* = *RT*.ln*K*_*d*_, and −*T*Δ*S* = Δ*G* − Δ*H*. Errors considered in the measured parameters (±20% in *K*_*d*_ and ±0.3 kcal/mol in Δ*H* and −*T*Δ*S*) were taken larger than the standard deviation between replicates and the numerical error after the fitting analysis.

## Data Availability

All data are contained within the manuscript and the supplementary materials.

## Abbreviations

^app^MW: Apparent molecular weight
BS^3^: Homobifunctional-bis[sulfosuccinimidyl]-suberate
CHCHD4: Coiled-coil-helix-coiled-coil-helix domain containing 4
CTC: Charge transfer complex
CD: Circular dichroism
CypA: Cyclophilin A
DCPIP: Dichlorophenolindophenol
FAD: Flavin adenine dinucleotide
hAIF: Human apoptosis-inducing factor
hAIF_ox_: Oxidized hAIF
hAIF_rd_: Reduced hAIF
hAIF_phrd_: Photoreduced hAIF
IMS: Intermembrane space
NADH: Reduced nicotinamide adenine dinucleotide
NAD^+^: Oxidized nicotinamide adenine dinucleotide
OXPHOS: Oxidative phosphorylation
PARP-1: Poly(ADP-ribose) polymerase-1
ROS: Reactive oxygen species
SAS: Solvent accessible surface.

## Supplementary Materials

Supplementary Materials The file contains the following: (i) the protocol for production and purification of proteins and for MD simulations and (ii) Figures S1-S9.
