# Supplementary material for: W196 and the β-Hairpin Motif Modulate the Redox Switch of Conformation and the Biomolecular Interaction Network of the Apoptosis-Inducing Factor
Source: Oxid Med Cell Longev. 2021 Jan 15;2021:6673661. doi: 10.1155/2021/6673661 (PMC7822688; doi:10.1155/2021/6673661)
Supplement: Supplementary Materials — The file contains the following: (i) the protocol for production and purification of proteins and for MD simulations and (ii) Figures S1-S9. [file 6673661.f1.zip › S1_W196_201101.pptx]

## Slide 1
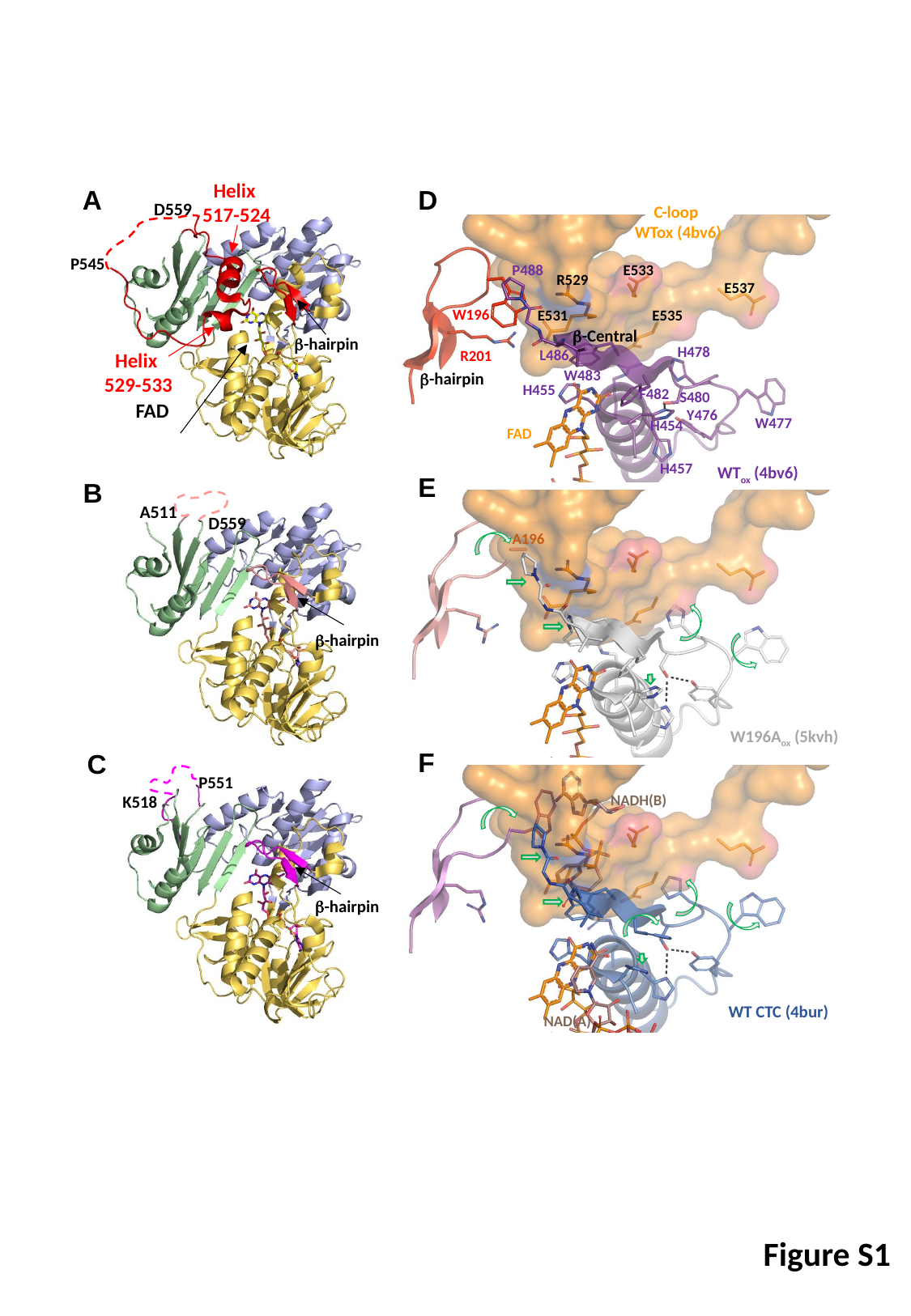

Helix
517-524
Helix
529-533
A
D559
P545
β-hairpin
FAD
D
C-loop
WTox (4bv6)
P488
E533
R529
E537
W196
E531
E535
β-Central
H478
L486
R201
W483
β-hairpin
H455
F482
S480
Y476
W477
H454
FAD
H457
WTox (4bv6)
A196
W196Aox (5kvh)
NADH(B)
WT CTC (4bur)
NAD(A)
E
B
A511
D559
β-hairpin
F
C
β-hairpin
P551
K518
Figure S1
