# Supplementary material for: W196 and the β-Hairpin Motif Modulate the Redox Switch of Conformation and the Biomolecular Interaction Network of the Apoptosis-Inducing Factor
Source: Oxid Med Cell Longev. 2021 Jan 15;2021:6673661. doi: 10.1155/2021/6673661 (PMC7822688; doi:10.1155/2021/6673661)
Supplement: Supplementary Materials — The file contains the following: (i) the protocol for production and purification of proteins and for MD simulations and (ii) Figures S1-S9. [file 6673661.f1.zip › S8_W196_201101.pptx]

## Slide 1
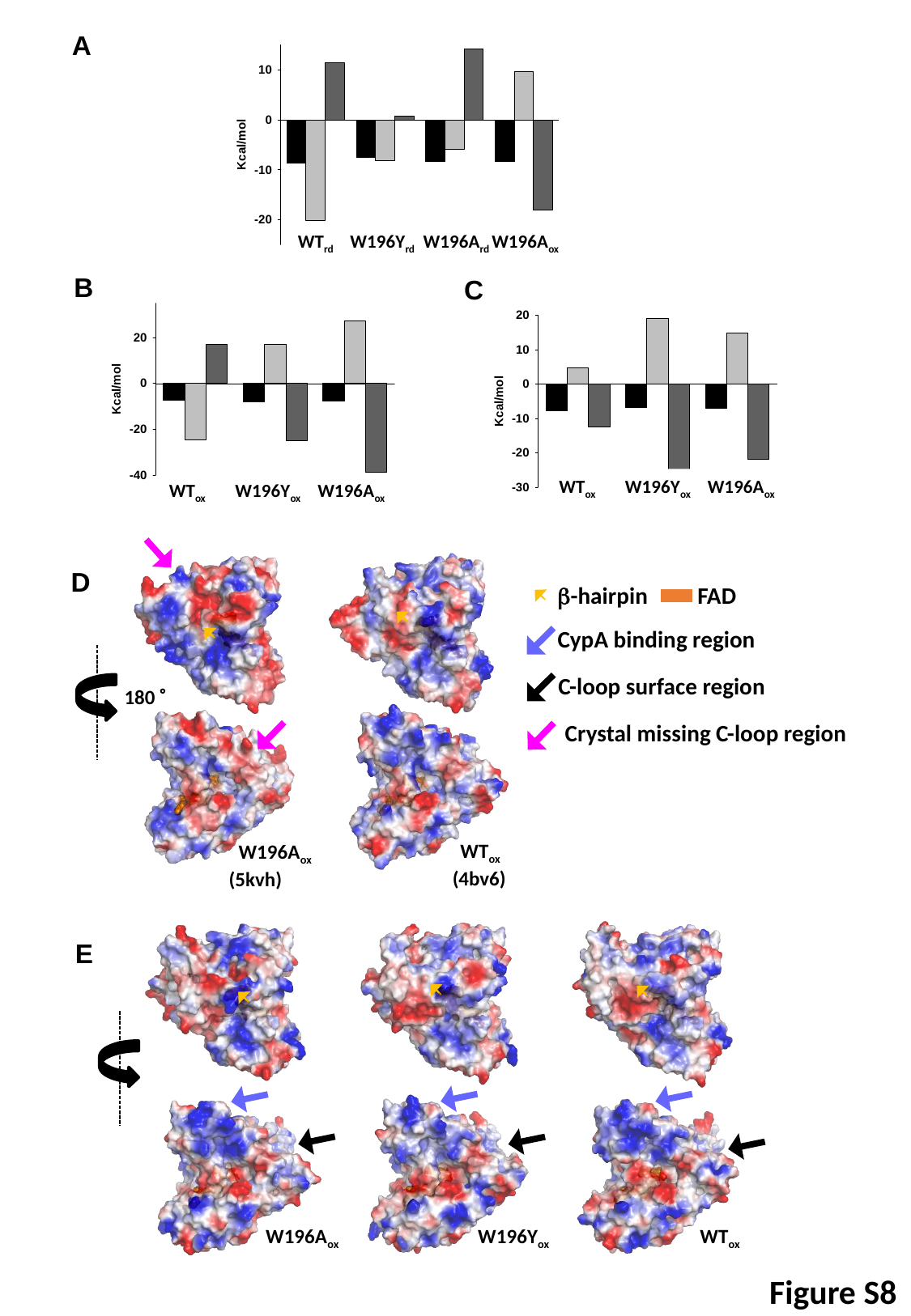

WTrd W196Yrd W196Ard W196Aox
A
B
C
WTox W196Yox W196Aox
W196Aox (5kvh)
WTox
(4bv6)
D
FAD
β-hairpin
CypA binding region
C-loop surface region
Crystal missing C-loop region
180 
W196Aox
WTox
W196Yox
E
WTox W196Yox W196Aox
Figure S8
