# Supplementary material for: W196 and the β-Hairpin Motif Modulate the Redox Switch of Conformation and the Biomolecular Interaction Network of the Apoptosis-Inducing Factor
Source: Oxid Med Cell Longev. 2021 Jan 15;2021:6673661. doi: 10.1155/2021/6673661 (PMC7822688; doi:10.1155/2021/6673661)
Supplement: Supplementary Materials — The file contains the following: (i) the protocol for production and purification of proteins and for MD simulations and (ii) Figures S1-S9. [file 6673661.f1.zip › S9_W196_201101.pptx]

## Slide 1
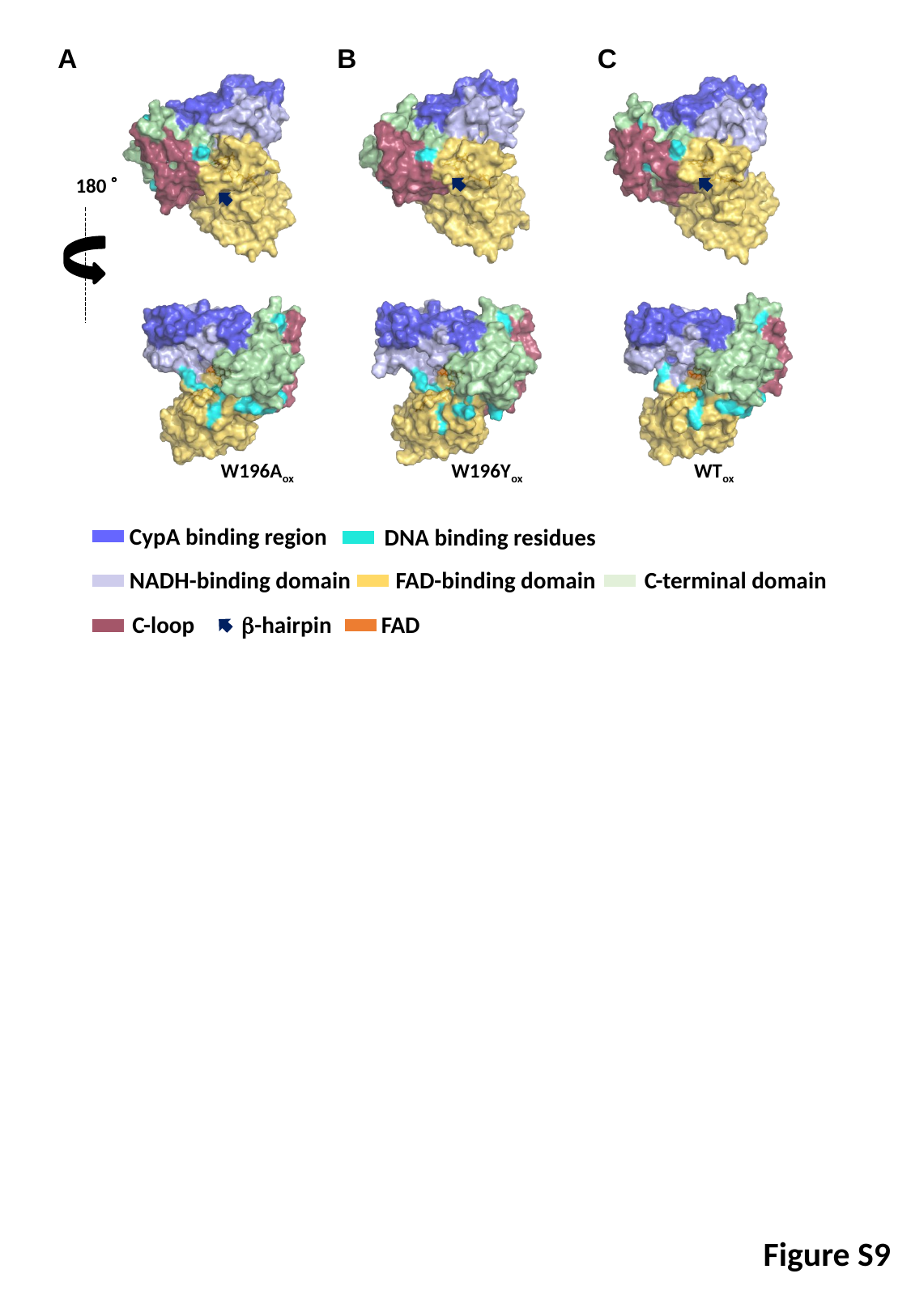

A
B
C
180 
WTox
W196Aox
W196Yox
CypA binding region
DNA binding residues
C-terminal domain
NADH-binding domain
FAD-binding domain
FAD
β-hairpin
C-loop
Figure S9
