# Supplementary material for: W196 and the β-Hairpin Motif Modulate the Redox Switch of Conformation and the Biomolecular Interaction Network of the Apoptosis-Inducing Factor
Source: Oxid Med Cell Longev. 2021 Jan 15;2021:6673661. doi: 10.1155/2021/6673661 (PMC7822688; doi:10.1155/2021/6673661)
Supplement: Supplementary Materials — The file contains the following: (i) the protocol for production and purification of proteins and for MD simulations and (ii) Figures S1-S9. [file 6673661.f1.zip › SI_W196_201215.docx]

**Supplementary materials**

**W196 and the β-hairpin motif modulate the redox switch of conformation and the biomolecular interaction network of the Apoptosis Inducing Factor**

Silvia Romero-Tamayo^a,b^, Ruben Laplaza^c,d^, Adrian Velazquez-Campoy^a,b,e,f,g^, Raquel Villanueva^a,b^, Milagros Medina^a,b*^ and Patricia Ferreira^a,b*^.

^a^Departamento de Bioquímica y Biología Molecular y Celular, Facultad de Ciencias, Universidad de Zaragoza, Spain.

^b^Instituto de Biocomputación y Física de Sistemas Complejos, BIFI (GBsC-CSIC and BIFI-IQFR Joint Units), Universidad de Zaragoza, Spain.

^c^Sorbonne Université, CNRS, Laboratoire de Chimie Théorique, LCT, 75005 Paris, France

^d^Departamento de Química Física, Universidad de Zaragoza, 50009 Zaragoza, Spain

^e^Fundación ARAID, Diputación General de Aragón, Spain.

^f^ Aragon Institute for Health Research (IIS Aragon), Zaragoza, Spain.

^g^ Biomedical Research Networking Centre for Liver and Digestive Diseases (CIBERehd), Madrid, Spain

^*^*Correspondence to*:

Dr. Milagros Medina. Departamento de Bioquímica y Biología Molecular y Celular. Facultad de Ciencias. Pedro Cerbuna 12. Universidad de Zaragoza. 50009-Zaragoza. Spain. Fax: +34 976 762123; Phone: +34 976 762476 e-mail: mmedina@unizar.es

Dr. Patricia Ferreira. Departamento de Bioquímica y Biología Molecular y Celular. Facultad de Ciencias. Pedro Cerbuna 12. Universidad de Zaragoza. 50009-Zaragoza. Spain. Fax: +34 976 762123; Phone: +34 876 553774 e-mail: ferreira@unizar.es.

**Supplementary Methods**

**Production and purification of proteins_-_** For the production of WT hAIF_∆1-101_ and its W196Y, W196L and W196A variants, the transformed bacteria were grown in 10 L LB medium containing 30 mg/L kanamycin and supplemented with 0.08 g/L of riboflavin and then incubated at 37ºC and 180 r.p.m. At OD_600nm_ ≈ 0.8, cultures were induced with 1 mM IPTG (*BioChemica*) and incubated at 25ºC and 100 r.p.m for additional 18 h. After that, cells were harvested and proteins were purified by following the procedure already described for the WT [1]. For CypA production, the transformed bacteria were grown in 10 L LB medium containing 30 mg/L kanamycin and incubated at 37ºC and 180 r.p.m. At A_600nm_ ≈ 0.5, protein expression was induced with 0.5 mM IPTG (*BioChemica*), and bacteria cultures were incubated for 3 additional hours. After that, cells were harvested by centrifugation and subsequently resuspended in 50 mM Tris/HCl, pH 8.0, NaCl 1mM and lysed by sonication on ice. The cell lysate was centrifuged to remove debris, and then was mixed with 5 mL of Ni^2+^ IMAC Sepharose 6 Fast Flow gel (*GE Healthcare*) previously equilibrated in the above buffer containing 4 mM imidazole. The mixture was incubated on a bidirectional orbital rocker for 1 h at 4 ºC, and then loaded into a column. The protein was eluted with a linear imidazole gradient from 40 to 250 mM with 10% glycerol in 50 mM Tris, pH 8.0, 150 mM NaCl. Protein fractions were pooled, concentrated and dialyzed with 50 mM potassium phosphate, pH 7.4. A similar protocol was used for CHCHD4 and H2AX with the differences noted below. For CHCHD4: i) bacteria cultures were induced using 1mM IPTG; ii) the imidazole gradient was from 40 to 1000 mM in 50 mM potassium phosphate pH 7.0, 150 mM NaCl; iii) protein was stored in 50 mM potassium phosphate, pH 7.4, with 10 mM DTT, to maintain the catalytic Cys53-Pro54-Cys55 disulfide motif in reduced state. For H2AX: i) bacteria cultures were induced at A_600nm_ ≈ 0.6 with 1mM IPTG. ii) the imidazole gradient was from 40 to 500 mM in 50 mM potassium phosphate pH 7.4, 150 mM NaCl; iii) protein was stored in 50 mM potassium phosphate pH 7.4 at -80 ºC.

**MD simulations-** Once the protein models were produced, cofactors or coenzymes were reintroduced and 50 ps MD simulations with Generalized-Born -Molecular Volume (GBMV) solvation [2] and CHARMM c39b1- were carried out to remove potential clashes. Protonation states were assessed using PROPKA 3.0 [3]; H131, H457 and H478 were ε-protonated, while H454 and H455 were δ-protonated. MD simulations were performed using CHARMM c39b1 and the charmm36 force field [4]. Parameters for FAD, FADH^-,^ NADH and NAD^-^ were generated using the CgenFF server [5] and density functional theory (DFT). DFT calculations were performed with the Gaussian09 rev.D01 package [6] at the B3LYP/def2-SVP+GD3BJ level with a water-like polarizable continuum model [6, 7]. A Monte-Carlo scheme was used for neutralization, adding ions to 150 mM. A time step of 2 fs, NVT conditions, and a TIP3P water model were used. A standard workflow routine including solvation, neutralization, minimization plus heating and equilibration over 200 ps was performed to the models, followed by a 10 ns MD production run at 298.15 K. Five replicas were performed for each structure. The CHARMM software analysis tools were used to evaluate the resulting conformational ensembles, using a spherical probe with 1.4 Å radius to calculate accessible surfaces. VMD [8] and PyMol [9] were used to analyze and to visualize structural data.

**Supplementary Figures**

**Figure S1. Comparative overview of the crystallographic structures of WT hAIF_Δ1-101ox_ (pdb 4BV6), W196A_ox_ hAIF_Δ1-101ox_ (pdb 5 KVH) and WT hAIF_Δ1-101rd_:2NAD(H)** **CTC (pdb 4BUR)**. Panels on the left show overall cartoon representations for (A) WT_ox_ (B) W196A_ox_, and (C) WT CTC. The FAD-, NADH-, and C-terminal domains are respectively colored in gold, light blue and pale green. FAD is drawn as sticks with C atoms in yellow, salmon and magenta respectively for WT_ox_, W196A_ox_ and WT CTC structures. The β-hairpin as well as visible residues in the regulatory C-loop (residues 509-560) are shown in red, salmon and magenta respectively for WT_ox_, W196A_ox_ and WT CTC structures. Missing fragments of the C-loop (P545-D559, A511-D559 and K518-G557 in WT_ox_ chain A, W196A_ox_ chain A and WT CTC chain C structures, respectively) are drawn as dashed lines. Panels on the right show a detail of the relationship of the β-hairpin with the central β-strand for (D) WT_ox_, (E) W196A_ox_, and (F) WT CTC. In all cases FAD is drawn as sticks with C atoms in orange. All panels show the regulatory C-loop as seen in the WT_ox_ structure (this fragment is missing for the other two structures) with an orange transparent surface. Coenzymes in the WT CTC structure are shown as CPK colored sticks with C atoms in pink. Green arrows indicate relevant displacements of structural elements relative to the crystallographic WT_ox_ structure.

**Figure S2. Spectral properties of the W196 hAIF_∆1-101ox_ variants.** (A) Visible absorption spectra of WT (16 µM), W196Y (39 µM), W196L (22 µM) and W196A (24 µM) hAIF_∆1-101ox_ variants. Spectra were recorded in 50 mM potassium phosphate, pH 7.4, at 25 ºC. Different protein concentrations were used for each variant to clarify the figure. Far-UV CD spectra of (B) the different variants (1 µM) and (C) mixtures of each variant (0.5 µM) with a 20-fold excess of NADH. Near-UV/Vis CD spectra of samples containing (D) hAIF_∆1-101ox_ variants (20 µM) and (E) mixtures of each variant (20 µM) with a 10-fold excess of NADH. CD spectra were recorded at 10 ºC in 50 mM potassium, pH 7.4, and at final ionic strength of 150 mM. In all panels: WT as solid black line, W196Y as dotted black line, W196L as dashed black line and W196A as grey solid line.

**Figure S3. Dynamics of the models of hAIF_∆1-101_ variants.** Averaged values of energy, radius of gyration (RyG), root mean square deviation (RMSD) of backbone, as well as solvent accessible surface (SAS) for the FAD cofactor and the NAD^+^/H coenzymes. (B) Averaged values of SAS for the overall protein, for the β–hairpin, and the predicted interaction surfaces for CypA (residues 345-397), and DNA (residue R265, R446, R449, R450, R451, K510, K518, R584, K590 and K593). All averaged values correspond to the mean of five MD replicates in the 2-10 ns range for each assayed condition. All dynamics equilibrated in the 0-2 ns range.

**Figure S4. Dynamics of the active site in hAIF_∆1-101_ variants.** Trajectories of selected distances at the active site environment in (A) hAIF_∆1-101ox_ and (B) CTC variants, as well as (C) between the flavin and nicotinamide reacting rings in CTC models. All panels represent the evolution of distances in five MD replicates run for each model. (D) Structural model of the hAIF_∆1-101_ active site with detail of distances represented in the different trajectories.

**Figure S5. Kinetic characterization of W196 hAIF_Δ1-101_ variants.** Spectral evolution of the reduction of the (A) W196Y (~10 µM) and (B) W196L (~ 10 µM) variants when mixed with NADH (5 mM). Spectra for the reduction of W196Y hAIF_∆1-101ox_ are shown at 0.001, 0.07, 0.09, 0.477, 0.99, 2, 17, 28, 43, 55 s after mixing and those of W196L at 0.025, 0.05, 0.075, 0.1, 0.15, 0.2, 0.35, 0.55, 0.9 s after mixing. Dotted lines correspond to the spectra of oxidized enzymes before mixing. The insets show the absorbance spectra for the intermediate species obtained by global fitting of the spectral evolution to a single step model (A🡪B) and the evolution of the concentration of each species. Time course of the absorbance changes at (C) the flavin band I (451 nm) and (D) the CTC band (700 nm) reduction by NADH of WT (black), W196Y (dashed line), W196L (dotted line) and W196A (grey) variants. Spectral evolution of CTC formation upon mixing of the hAIF_∆1-101phrd_ forms of (F) W196Y (~8 µM) and (G) W196L (~10 µM) with NAD^+^ (5 mM) under anaerobic conditions. Spectra for CTC formation of W196Y are shown at 0.002, 0.004, 0.006, 0.008, 0.01, 0.02, 0.03, 0.05, 0.07, 0.1, 0.2, 0.5, 0.7, 1, 1.5, 2 s after mixing; and those of W196L at 0.001, 0.002,0.003, 0.006, 0.007, 0.01, 0.02, 0.03, 0.04, 0.05, 0.06, 0.07, 0.08, 0.09, 0.1, 0.2, 0.3, 1 and 2 s after mixing. The insets show the absorbance evolution at 750 nm and the fits at this wavelength when globally fitting evolution at a single step model (A🡪B). Assays were carried out in a stopped-flow spectrophotometer in 50 mM potassium phosphate, pH 7.4, and at 25 ºC. All indicated concentrations are final ones.

**Figure S6. Dynamics of selected distances at the core of hAIF_∆1-101_ variants.** Time evolution of selected distances involving (A) the H478 residue, (B) the Ser480 residue and (C) the interactions of negatively charged residues of the C-loop with the protein core. For each variant data show averaged values for the 5 MD replicates run for each model in the hAIF_∆1-101ox_ (bold lines) and CTC (line) states.

**Figure S7. Effect of W196 mutations on the binding to CypA and dsDNA to hAIF_Δ1-101_.** Thermograms (upper panels) and binding isotherms with integrated heat (lower panels) for the calorimetric titration of (A) WT, (B) W196Y, and (C) W196A hAIF_Δ1-101ox_ with CypA and of (B) WT, (C) W196Y, and (D) W196A hAIF_Δ1-101ox_ with dsDNA. Titrations were carried out in 50 mM potassium phosphate, pH 7.4, at 25 ºC.

**Figure S8. Thermodynamic dissection of the interaction of the different hAIF_Δ1-101_ variants with interacting partners.** (A) CHCHD4, (B) CypA and (C) DNA. Measurements were carried out in 50 mM potassium phosphate, pH 7.4, at 15 ºC for DNA and 25 ºC for protein partners. The Gibbs energy (ΔG), enthalpy (ΔH), and entropy (−TΔS) contributions to the binding are indicated in black, light grey and dark grey bars, respectively. (D) Comparison of the overall electrostatic potential surfaces of the 3D crystallographic structures of W196A_ox_ and WT_ox_. Positions were the C-loop is missing are particularly highlighted by a pink arrow. (E) Comparison of the overall electrostatic potential surfaces of final structures for representative replicates of the MD simulations for the modeled structures of W196A_ox_, W196Y_ox_ and WT_ox_. The interaction region predicted for CypA binding are particularly highlighted by a violet arrow.

**Figure S9. Dynamics of the interaction surfaces for partners in hAIF_∆1-101_ variants.** Comparative overview of surfaces for equilibrated structures for (A) W196A_ox_, (B) W196Y_ox_, (C) WT_ox_. Color codes of surfaces of interaction and of ligands are as indicated below the figure. Ligands are also shown as sticks within the surfaces. Each figure shows the final structure of one representative replicate of the MD production.

**References**

1. Ferreira, P., Villanueva, R., Martínez-Júlvez, M., Herguedas, B., Marcuello, C., Fernandez-Silva, P., Cabon, L., Hermoso, J. A., Lostao, A., Susin, S. A. & Medina, M. (2014) Structural insights into the coenzyme mediated monomer-dimer transition of the pro-apoptotic apoptosis inducing factor, *Biochemistry.* **53**, 4204-15.

2. Lee, M. S. & Salsbury, F. R. J. (2002) Novel generalized Born methods in pp. 10606The Journal of Chemical Physics.

3. Olsson, M. H., Søndergaard, C. R., Rostkowski, M. & Jensen, J. H. (2011) PROPKA3: Consistent Treatment of Internal and Surface Residues in Empirical pKa Predictions, *J Chem Theory Comput.* **7**, 525-37.

4. Brooks, B. R., Brooks, C. L., Mackerell, A. D., Nilsson, L., Petrella, R. J., Roux, B., Won, Y., Archontis, G., Bartels, C., Boresch, S., Caflisch, A., Caves, L., Cui, Q., Dinner, A. R., Feig, M., Fischer, S., Gao, J., Hodoscek, M., Im, W., Kuczera, K., Lazaridis, T., Ma, J., Ovchinnikov, V., Paci, E., Pastor, R. W., Post, C. B., Pu, J. Z., Schaefer, M., Tidor, B., Venable, R. M., Woodcock, H. L., Wu, X., Yang, W., York, D. M. & Karplus, M. (2009) CHARMM: the biomolecular simulation program, *J Comput Chem.* **30**, 1545-614.

5. Vanommeslaeghe, K., Hatcher, E., Acharya, C., Kundu, S., Zhong, S., Shim, J., Darian, E., Guvench, O., Lopes, P., Vorobyov, I. & Mackerell, A. D. (2010) CHARMM general force field: A force field for drug-like molecules compatible with the CHARMM all-atom additive biological force fields, *J Comput Chem.* **31**, 671-90.

6. Frisch, M. J., G. W. Trucks, H. B. Schlegel, G. E. Scuseria, A., M., Robb, J. R. Cheeseman, G. Scalmani, V., Barone, G. A. P., H. Nakatsuji, M. C. X. Li, A. Marenich, J., B., B. G. Janesko, R. Gomperts, B. Mennucci, H. P. Hratchian, J. V. Ortiz, A. F. Izmaylov, J. L. Sonnenberg, D., Williams-Young, F. Ding, F. L., F., E., J. Goings, B. Peng, A., Petrone, T. Henderson, D. Ranasinghe, V. G., Z., J. Gao, N. Rega, G., Zheng, W. Liang, M. Hada, M. Ehara, K. Toyota, R. Fukuda, J. Hasegawa, M., Ishida, T. Nakajima, Y. Honda, O. Kitao, H. Nakai, T. Vreven, K. Throssell, J., J. A. Montgomery, J. E. Peralta, F. Ogliaro, M., B., J. J. Heyd, E., Brothers, K. N. Kudin, V. N. Staroverov, T. Keith, R., K., J. Normand, K. Raghavachari, A. Rendell, J. C. Burant, S. S., I., J. Tomasi, M. Cossi, J. M. Millam, M. Klene, C. Adamo, R. Cammi, J. W., O., R. L. Martin, K., Morokuma, O. Farkas, Foresman, J. B. & Fox, D. J. (2016) Gaussian 09, Revision  A.02 in Gaussian, Inc.,  Wallingford CT.

7. Grimme, S., Ehrlich, S. & Goerigk, L. (2011) Effect of the damping function in dispersion corrected density functional theory, *J Comput Chem.* **32**, 1456-65.

8. Humphrey, W., Dalke, A. & Schulten, K. (1996) VMD: visual molecular dynamics, *J Mol Graph.* **14**, 33-8, 27-8.

9. Delano, W. L. (2002) PyMOL: an open-source molecular graphics tool, *CCP4 Newsletter On Protein Crystallography.* **40**, 82-92.
