# Supplementary figures and images for: W196 and the β-Hairpin Motif Modulate the Redox Switch of Conformation and the Biomolecular Interaction Network of the Apoptosis-Inducing Factor
Source: Oxid Med Cell Longev. 2021 Jan 15;2021:6673661. doi: 10.1155/2021/6673661 (PMC7822688; doi:10.1155/2021/6673661)

## Slide 1
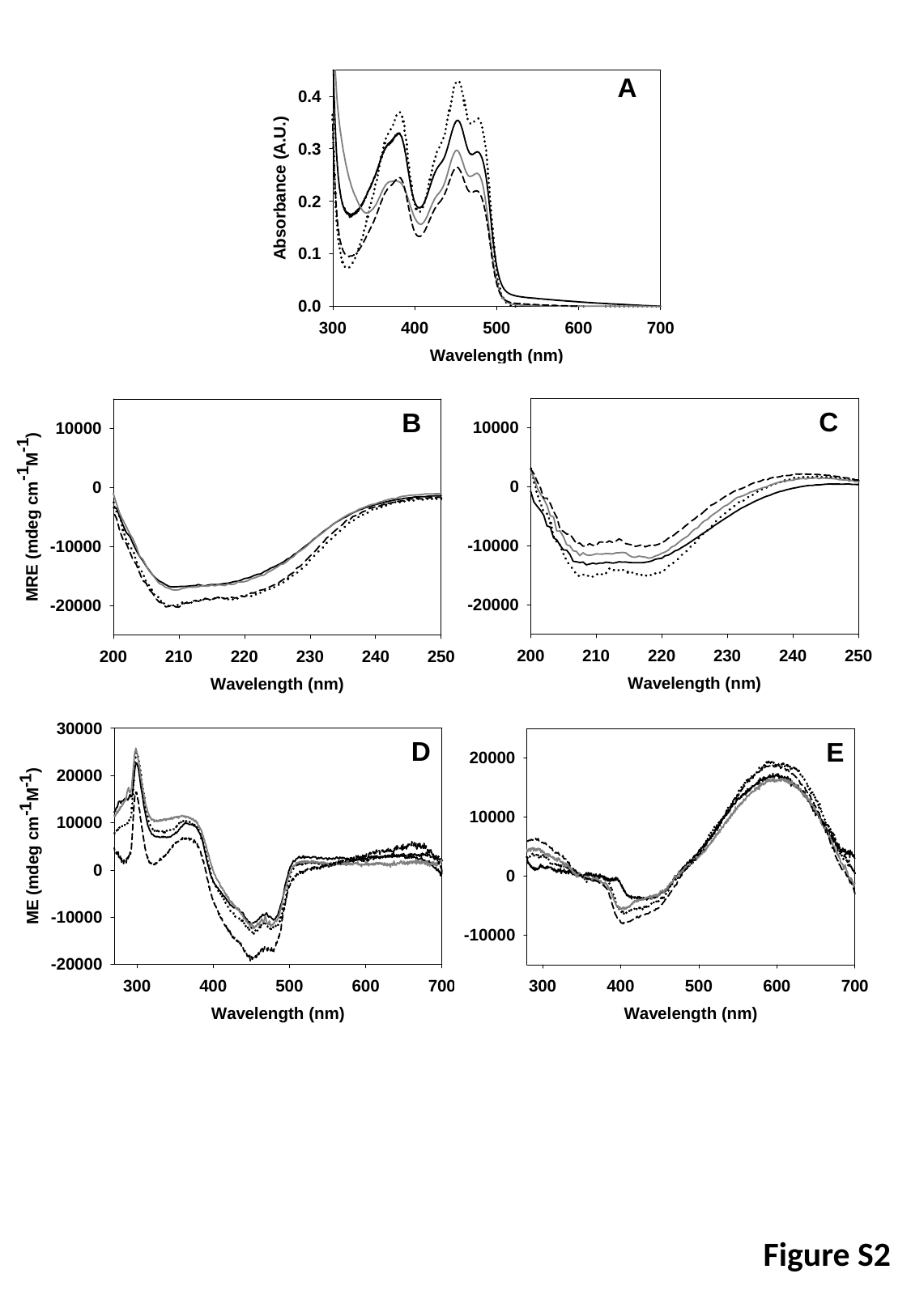

A
C
B
D
E
Figure S2

Supplement: Supplementary Materials — The file contains the following: (i) the protocol for production and purification of proteins and for MD simulations and (ii) Figures S1-S9. [file 6673661.f1.zip › S2_W196_201101.pptx]

## Slide 1
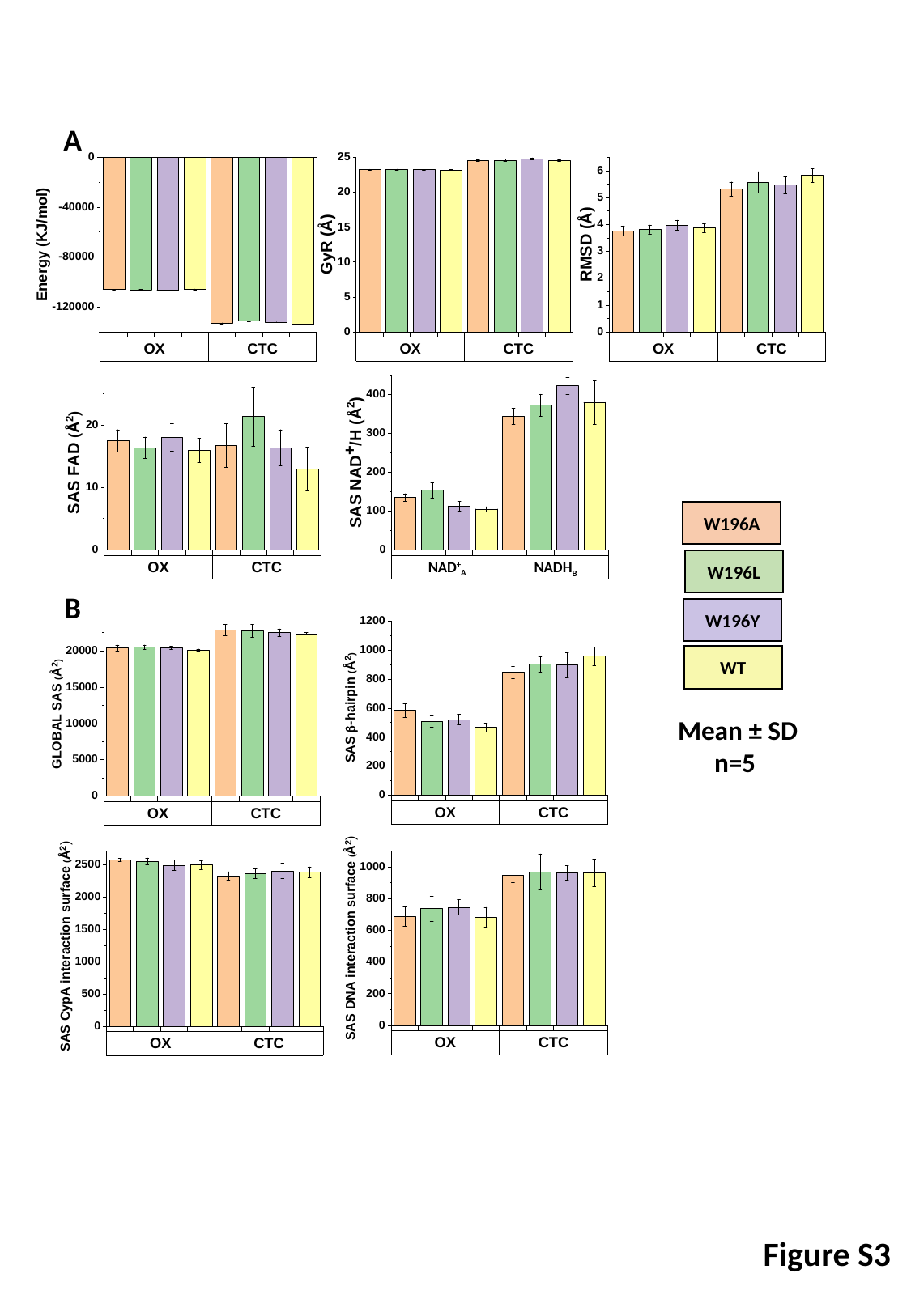

A
NAD+A
NADHB
W196A
W196L
W196Y
WT
B
Mean ± SD
n=5
Figure S3

Supplement: Supplementary Materials — The file contains the following: (i) the protocol for production and purification of proteins and for MD simulations and (ii) Figures S1-S9. [file 6673661.f1.zip › S3_W196_201101.pptx]

## Slide 1
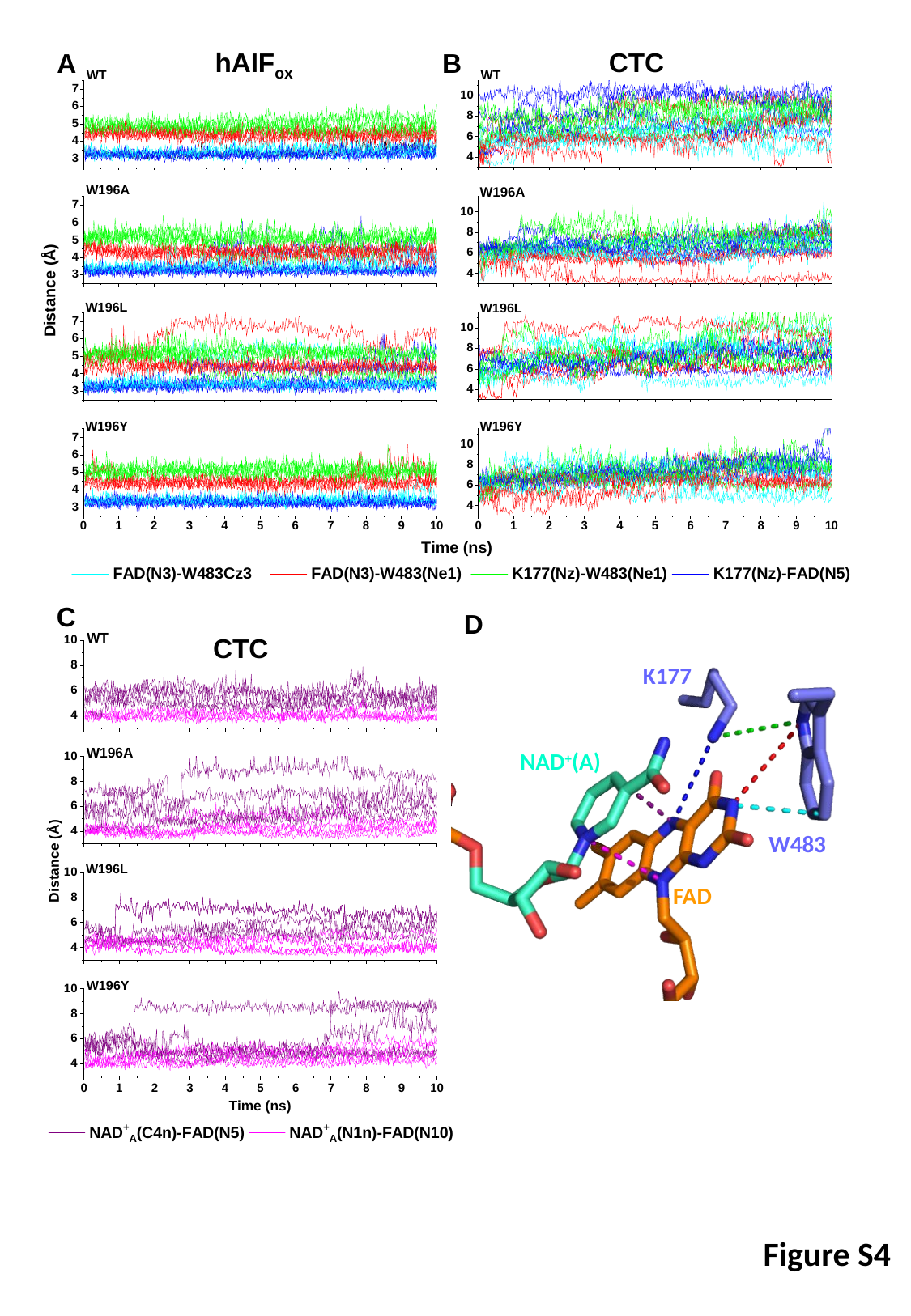

hAIFox
CTC
A
B
C
D
CTC
K177
NAD+(A)
W483
FAD
Figure S4

Supplement: Supplementary Materials — The file contains the following: (i) the protocol for production and purification of proteins and for MD simulations and (ii) Figures S1-S9. [file 6673661.f1.zip › S4_W196_201101.pptx]

## Slide 1
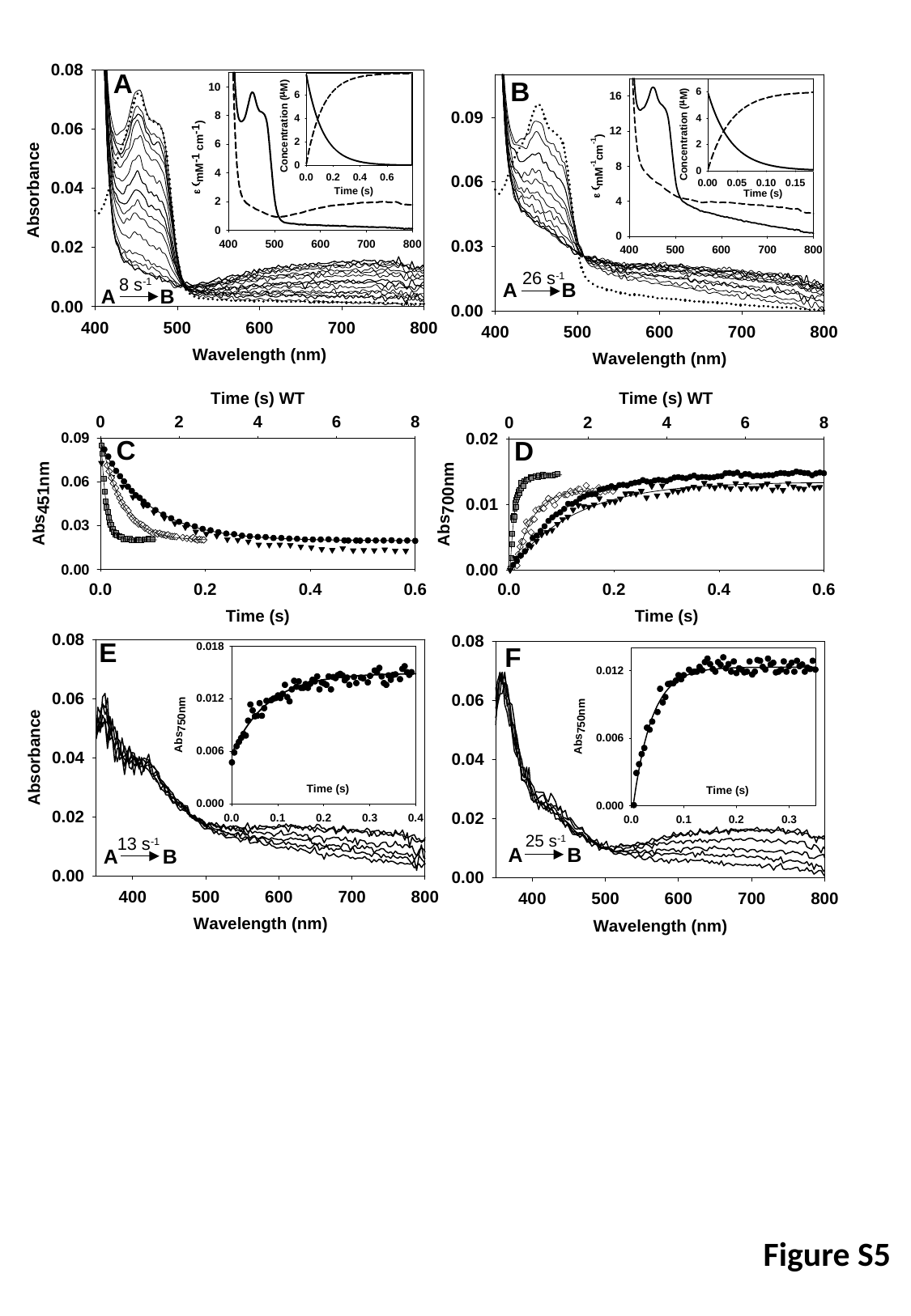

A
B
26 s-1
A B
8 s-1
A B
C
D
E
F
25 s-1
A B
13 s-1
A B
Figure S5

Supplement: Supplementary Materials — The file contains the following: (i) the protocol for production and purification of proteins and for MD simulations and (ii) Figures S1-S9. [file 6673661.f1.zip › S5_W196_201101.pptx]

## Slide 1
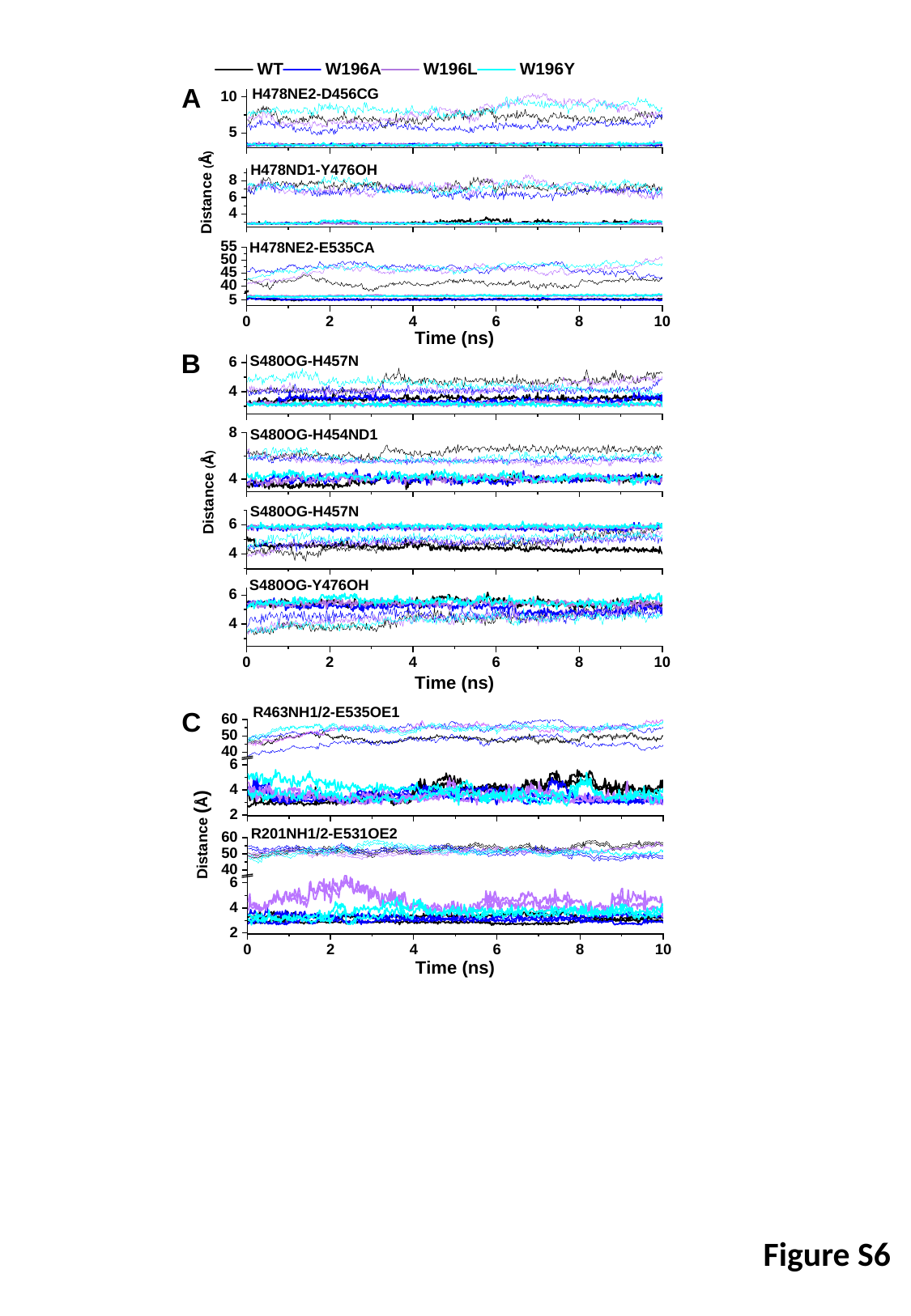

A
B
C
Figure S6

Supplement: Supplementary Materials — The file contains the following: (i) the protocol for production and purification of proteins and for MD simulations and (ii) Figures S1-S9. [file 6673661.f1.zip › S6_W196_201101.pptx]

## Slide 1
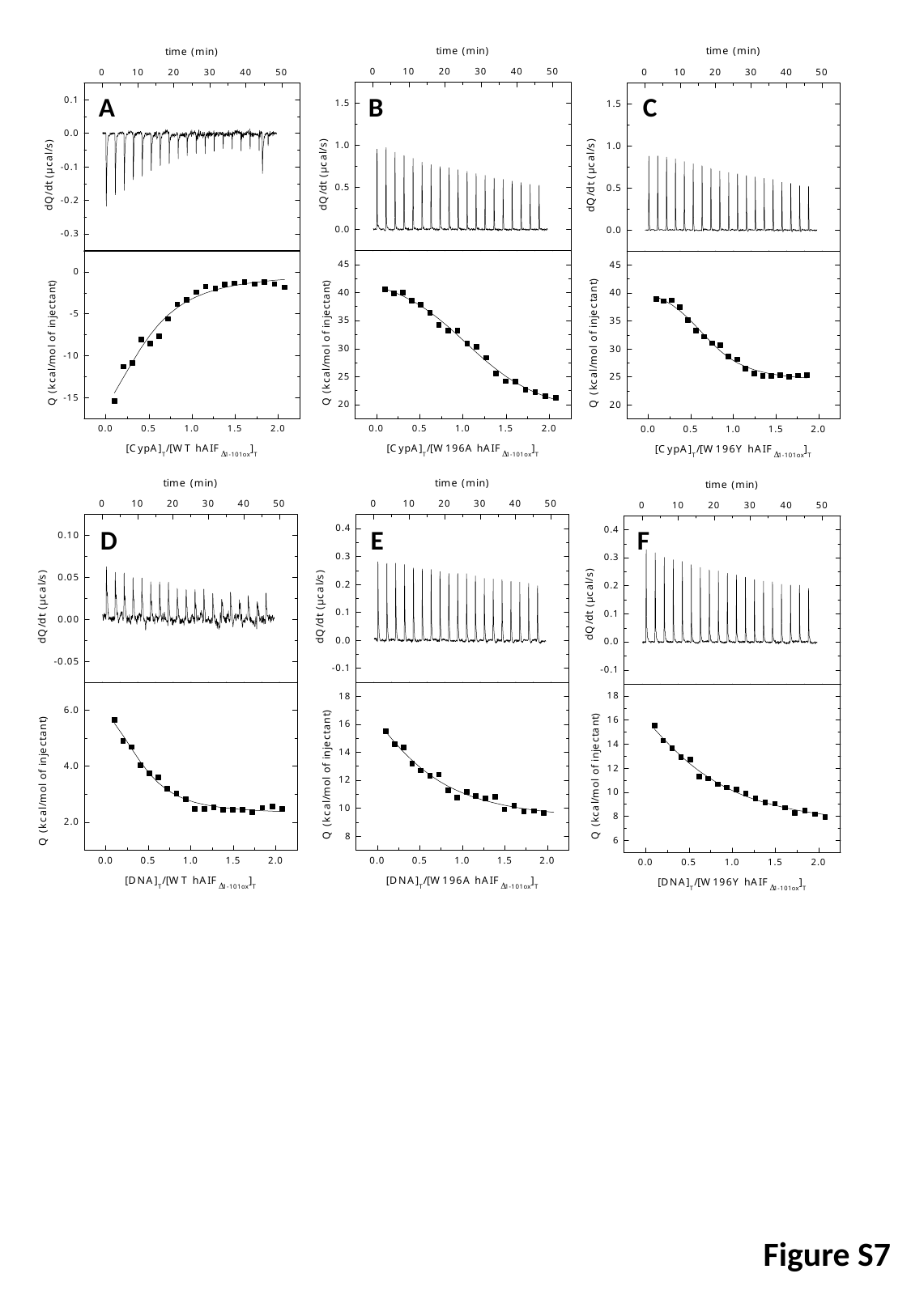

A
B
C
D
F
E
Figure S7

Supplement: Supplementary Materials — The file contains the following: (i) the protocol for production and purification of proteins and for MD simulations and (ii) Figures S1-S9. [file 6673661.f1.zip › S7_W196_201101.pptx]
